# The mitochondrial protein Sideroflexin 3 (SFXN3) influences neurodegeneration pathways *in vivo*

**DOI:** 10.1111/febs.16377

**Published:** 2022-02-06

**Authors:** Leire M. Ledahawsky, Maria Eirini Terzenidou, Ruairidh Edwards, Rachel A. Kline, Laura C. Graham, Samantha L. Eaton, Dinja van der Hoorn, Helena Chaytow, Yu‐Ting Huang, Ewout J. N. Groen, Anna A. L. Motyl, Douglas J. Lamont, Kostas Tokatlidis, Thomas M. Wishart, Thomas H. Gillingwater

**Affiliations:** ^1^ Edinburgh Medical School Biomedical Sciences University of Edinburgh UK; ^2^ Euan MacDonald Centre for Motor Neuron Disease Research University of Edinburgh UK; ^3^ 3526 Institute of Molecular Cell and Systems Biology College of Medical, Veterinary and Life Sciences University of Glasgow UK; ^4^ The Roslin Institute and R(D)SVS University of Edinburgh UK; ^5^ 3042 FingerPrints Proteomics Facility University of Dundee UK; ^6^ Department of Neurology and Neurosurgery UMC Utrecht Brain Center The Netherlands

**Keywords:** mitochondria, neurodegeneration, Parkinson's disease, Sideroflexin 3, synapse

## Abstract

Synapses are a primary pathological target in neurodegenerative diseases. Identifying therapeutic targets at the synapse could delay progression of numerous conditions. The mitochondrial protein SFXN3 is a neuronally enriched protein expressed in synaptic terminals and regulated by key synaptic proteins, including α‐synuclein. We first show that SFXN3 uses the carrier import pathway to insert into the inner mitochondrial membrane. Using high‐resolution proteomics on *Sfxn3*‐KO mice synapses, we then demonstrate that SFXN3 influences proteins and pathways associated with neurodegeneration and cell death (including CSPα and Caspase‐3), as well as neurological conditions (including Parkinson's disease and Alzheimer’s disease). Overexpression of SFXN3 orthologues in *Drosophila* models of Parkinson's disease significantly reduced dopaminergic neuron loss. In contrast, the loss of SFXN3 was insufficient to trigger neurodegeneration in mice, indicating an anti‐ rather than pro‐neurodegeneration role for SFXN3. Taken together, these results suggest a potential role for SFXN3 in the regulation of neurodegeneration pathways.

## Graphical abstract

SFXN3 is a neuronally enriched mitochondrial protein found at the synapse. Here, we show that SFXN3 influences expression levels of neurodegeneration and cell death‐associated proteins in mice, that its overexpression provides neuroprotection at the synapse in Drosophila models of Parkinson's disease, but that its absence or downregulation is insufficient to trigger neurodegeneration in mice and Drosophila.

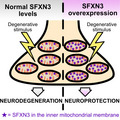

**Abbreviations**
AAC: ADP/ATP carrier
AD: Alzheimer’s disease
ALS: amyotrophic lateral sclerosis
BD: Batten’s disease
CCCP: carbonyl cyanide m‐chlorophenyl hydrazone
CFV: cresyl fast violet
CHCHD3: coiled‐coil‐helix‐coiled‐coil‐helix domain‐containing protein 3
CSPα: cysteine string protein α
DMEM: Dulbecco’s modified Eagle medium
FASP: filter‐aided sample preparation
FBS: fetal bovine serum
HD: Huntington’s disease
HEK: human embryonic kidney
IM: inner membrane
IMS: intermembrane space
IPA: ingenuity pathway analysis
LC‐MS/MS: liquid chromatography‐tandem mass spectrometry
MIB: mitochondria isolation buffer
OCT: optimal cutting temperature compound
OM: outer membrane
OXPHOS: oxidative phosphorylation
PD: Parkinson's disease
PFA: paraformaldehyde
SBTI: soybean trypsin inhibitor
SFXN3: Sideroflexin 3
*Sfxn3*‐KO: Sideroflexin 3 knockout
SMA: spinal muscular atrophy
Su9‐DHFR: subunit 9 of the *Neurospora crassa* ATP synthase fused to mouse dihydrofolate reductase
TCA: trichloroacetic acid
Tim22ts: Tim22 temperature sensitive
Tim9ts: Tim9 temperature sensitive
TMT: tandem mass tag
UAS: upstream activating sequence
Wld^S^
: Wallerian degeneration slow
WT: wild‐type
ΔTom70: Tom70‐depleted

## Introduction

Synapses are a major pathological target in many neurodegenerative diseases, including; Alzheimer’s disease (AD), Parkinson's disease (PD), Huntington’s disease (HD), amyotrophic lateral sclerosis (ALS), spinal muscular atrophy (SMA) and Batten’s disease (BD) [1, 2, 3]. Importantly, structural and functional modifications at the synapse precede neuronal death in the majority of these neurodegenerative conditions. For example, structural and functional changes at the synapse include reduced spine density in AD [4], impaired synaptic vesicle endocytosis and loss of striatal dopaminergic terminals in PD [5, 6] and defective inhibitory and excitatory synaptic transmission in Batten’s disease [7]. These findings highlight the importance of targeting pathways involved in driving synaptic pathology when designing strategies to delay the onset or progression of neurodegeneration *in vivo*.

α‐Synuclein is one well‐known modulator of synaptic pathology [8]. However, α‐synuclein is an intrinsically disordered protein, hence a difficult protein to target for therapeutic purposes [9]. Amorim et al. [10] identified Sideroflexin 3 (SFXN3) as a downstream target of α‐synuclein in synapses worthy of further investigation. SFXN3 belongs to a family of mitochondrial transmembrane proteins, including Sideroflexins 1–5, and is thought to play a role in iron transport [11, 12], although a recent study suggested that SFXN1 (and possibly also SFXN3) can also function as a serine transporter [13]. Interestingly, previous studies have identified a putative link between SFXN3 and neurodegenerative diseases, as SFXN3 mRNA and protein levels were found to be dysregulated in animal models and patients with PD [14, 15, 16]. Furthermore, it has recently been reported that SFXN3 is important for retinal homeostasis [17]. However, despite the identification of SFXN3 as a neuronally enriched protein that can be dysregulated in conditions such as PD, the contribution of SFXN3 to neurodegeneration pathways remains unexplored.

In this study, we confirm that SFXN3 follows the carrier import pathway to localise to the inner mitochondrial membrane (including in human cells). We demonstrate that experimental manipulation of SFXN3 impacts on expression levels of several known regulators of synaptic degeneration (including cysteine string protein α (CSPα) and caspase‐3) and controls expression of the constituents of established molecular neurodegeneration pathways associated with neurodegenerative diseases including PD and AD. Consistent with these findings, we show that increasing levels of *Sfxn3* orthologues is capable of influencing dopaminergic cell loss in *Drosophila* models of PD *in vivo*.

## Results and Discussion

### SFXN3 is an inner mitochondrial membrane protein that follows the carrier import pathway and is dependent on the inner membrane potential

**Fig. 1:**
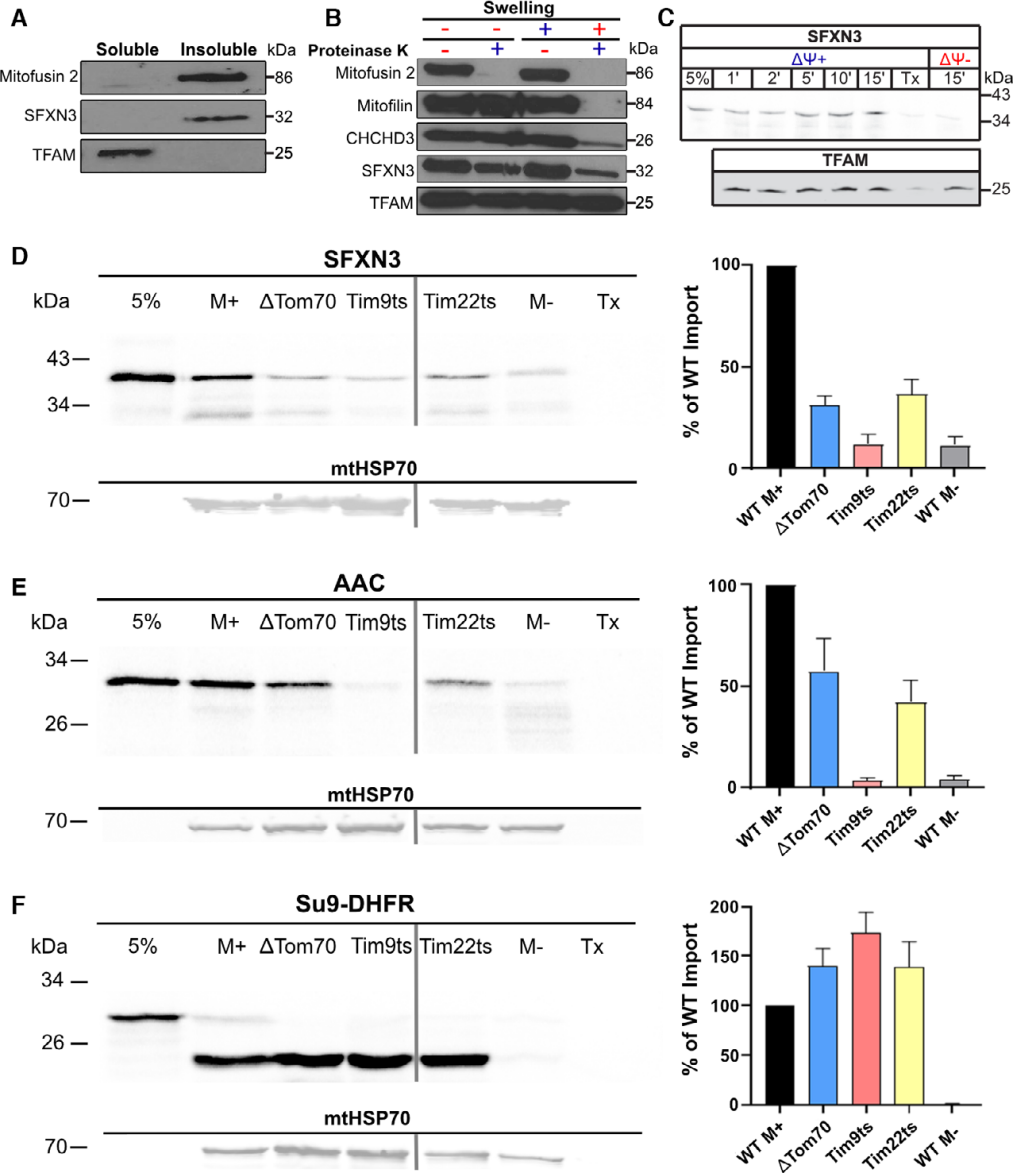
SFXN3 localisation in HEK293 mitochondria and import pathway in yeast mitochondria. (A) Immunoblotting of soluble fraction and insoluble fractions of HEK293 mitochondria following sodium carbonate extraction (*N* = 3). (B) Immunoblotting of HEK293 mitochondria and mitoplasts prepared by hypotonic swelling. Mitofusin 2 is an outer mitochondrial membrane protein, Mitofilin is an intermembrane space protein, CHCHD3 is an inner mitochondrial membrane protein and TFAM is a soluble matrix protein (*N* = 3). (C) ^35^S‐labelled SFXN3 was imported into isolated wild‐type HEK293 mitochondria in the presence or absence of the inner membrane potential (ΔΨ+) for the indicated time points followed by trypsin digestion to remove unimported material. Samples were run on SDS‐PAGE (*N* = 2). (D–F) Immunoblots of ^35^S‐labelled SFXN3, AAC, and ATP Synthase subunit 9 (Su9‐DHFR) imported into various control and mutant yeast mitochondria. mtHSP70 was probed as a housekeeping gene at 70 kDa. Quantification of import across yeast strains is shown as the percentage of WT M+ import. Data are shown as mean percentage ± SEM (*N* = 3). 5% = 5% of protein loaded into all other lanes. M+ = import under the presence of membrane potential. ΔDTom70 = Delta Tom70‐depleted. Tim9ts = Tim9 temperature sensitive. Tim 22ts = Tim22 temperature sensitive. M‐ = import into mitochondria without membrane potential. Tx = mitochondria solubilised with Triton‐X100. Where different parts of the gels were assembled in a single panel, the separation between the different gels is indicated by a perpendicular line (panels D, E and F).

The current literature concerning SFXN3 remains limited, with much remaining unknown with regard to its biochemical properties, as well as molecular and cellular functions *in vivo*. Therefore, we first set out to better characterise the localisation and biochemical properties of SFXN3. Amorim et al. [10] demonstrated that SFXN3 putatively localises to the inner mitochondrial membrane in mice. To confirm this localisation of SFXN3, and establish whether it is conserved across multicellular organisms, we performed localisation experiments using mitochondria from wild‐type, human HEK293 cells. Mitochondria underwent sodium carbonate extraction, a procedure that can efficiently distinguish soluble from membrane‐integrated proteins [18]. As expected, TFAM, a soluble matrix protein that is attached to mitochondrial DNA in the matrix functioning as a mitochondrial transcription factor [19, 20], was present in the soluble fraction, and Mitofusin 2, an outer mitochondrial membrane protein integrated into the outer membrane with two transmembrane segments [21], was present in the insoluble fraction. Importantly, SFXN3 was also present in the insoluble fraction, along with Mitofusin 2, confirming SFXN3 as a mitochondrial membrane‐integrated protein (Fig. 1A).

To further investigate the submitochondrial localisation of SFXN3, we performed a swelling and proteinase K protection assay. In this assay, swelling of the mitochondria results in selective opening of the outer membrane (OM) rendering proteins in the intermembrane space (IMS) and the inner membrane (IM) accessible to the proteinase K, whilst proteins in the matrix remain resistant to proteolysis since the IM remains sealed. In the first two lanes in Fig. 1B (where mitochondria were not swollen and kept in an isotonic buffer) addition of external proteinase K degraded only Mitofusin 2, which is an OM protein with a large domain exposed to the outer mitochondrial surface. By contrast, upon swelling of the mitochondria with a hypotonic buffer, SFNX3 followed exactly the same behaviour as mitochondrial IM proteins like coiled‐coil‐helix‐coiled‐coil‐helix domain‐containing protein 3 (CHCHD3) [22], and Mitofilin [23] (Fig. 1B). In agreement with the carbonate extraction results in Fig. 1A, a matrix protein (TFAM) behaves completely differently from SFXN3. We see that TFAM is stable against proteinase K treatment even upon swelling, confirming that the inner membrane remained intact in this treatment. The data of Fig. 1A,B therefore confirm that SFXN3 is an inner mitochondrial membrane protein in human cells, demonstrating that mitochondrial localisation of SFXN3 is conserved across humans and mice. Additionally, we wanted to assess the importance of the inner membrane potential on the import of SFXN3 into HEK293 mitochondria. When ^35^S‐labelled SFXN3 was presented to mitochondria in the absence of a membrane potential, it did not undergo mitochondrial import. However, in the presence of a membrane potential, SFXN3 was imported in increasing quantities over longer timepoints (Fig. 1C). This demonstrates the dependence of SFXN3 on the membrane potential for successful import into mammalian mitochondria.

Next, since SFXN3 is a nuclear‐encoded protein [24], we wanted to investigate the mechanism by which SFXN3 is imported into mitochondria. To do so, we used the TNT^®^ Coupled Reticulocyte Lysate System to obtain *in vitro* translated, radioactively labelled (with ^35^S‐Methionine) SFXN3, ADP/ATP carrier protein (AAC), and the hybrid precursor protein consisting of the presequence region 1‐64 of Subunit 9 of the *Neurospora crassa* ATP Synthase fused to mouse dihydrofolate reductase (Su9‐DHFR) protein products [25]. These radioactively labelled proteins in their native form were used to perform *in vitro* import into wild‐type and mutant *S. cerevisiae* mitochondria. The results illustrated in Fig. 1D–F show that specific mitochondrial *S. cerevisiae* mutations in components that are critical for the import of proteins following the metabolite carrier import pathway affect the mitochondrial import of SFXN3. Proteins that follow the carrier import pathway interact with the Tom70 receptor in the OM first, pass through the general import channel of Tom40 into the IMS where they are then specifically escorted in the aqueous IMS by the small Tim chaperones onto the inner membrane‐embedded Tim22 complex where they are inserted [26]. Specifically, mitochondria with lower levels of Tom70 (the main outer membrane receptor for the carrier pathway), or the IMS chaperone Tim9, or the Tim22 membrane insertion translocase, all affected the import of SFXN3. As a control, the AAC (a well‐characterised substrate of the carrier import pathway) was similarly affected. In contrast, the import of the matrix‐targeted Su9‐DHFR protein that follows the presequence pathway [27, 28] was clearly unaffected. These data therefore suggest that SFXN3 follows the carrier import pathway in mitochondria.

**Fig. 2:**
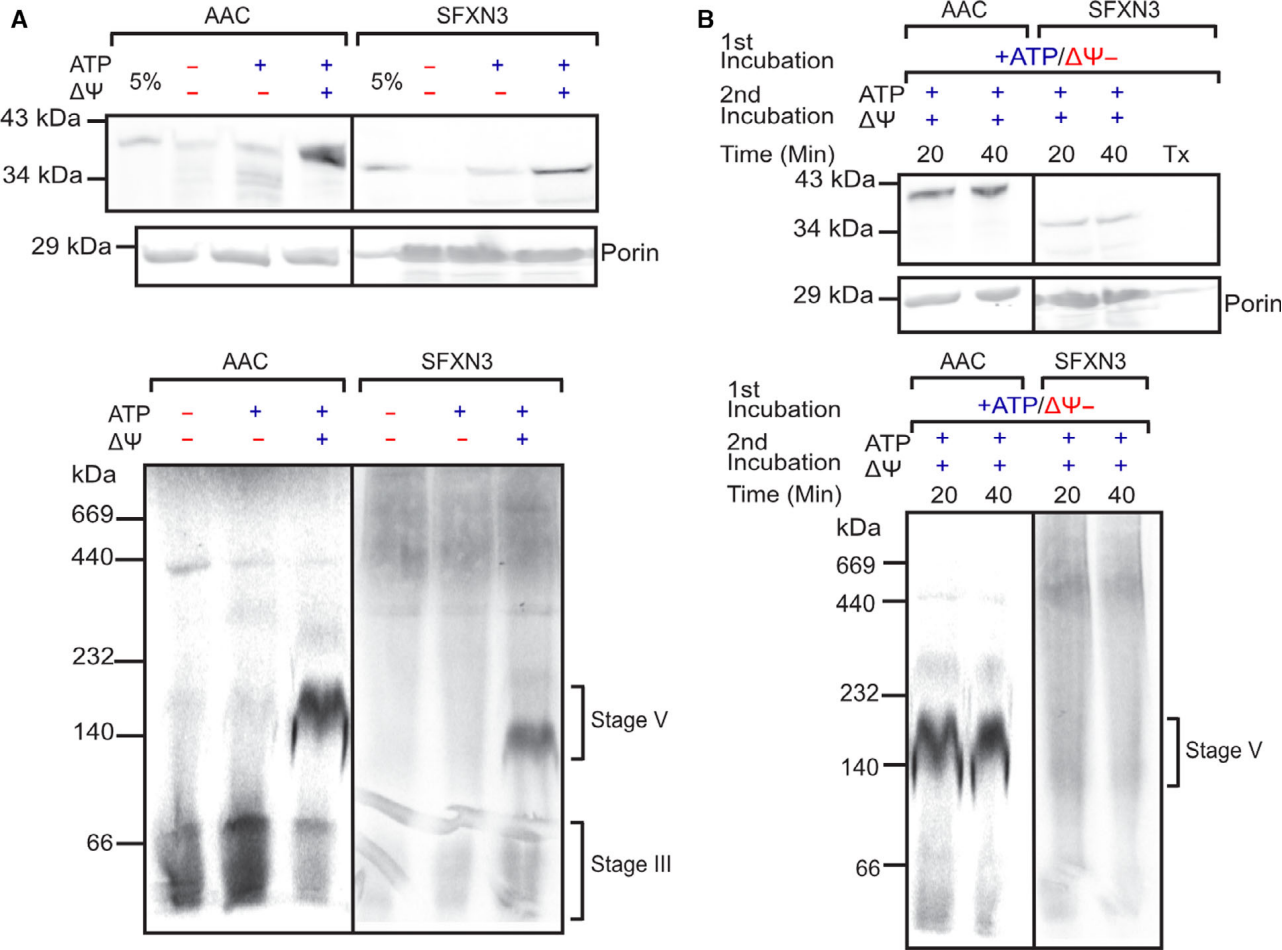
SFXN3 is imported similar to the AAC import pathway in a ΔΨ and ATP dependent manner. (A) ^35^S‐labelled AAC or SFXN3 were imported into wild‐type yeast mitochondria in the presence or absence of ΔΨ and ATP before being subject to SDS‐PAGE or BN‐PAGE. (B) ^35^S‐labelled AAC or SFXN3 were arrested in stage III of the carrier import pathway (ΔΨ−) for 10 min followed by re‐isolation and import in the presence of membrane potential (ΔΨ+) for the indicated timepoints. Samples were then subject to SDS‐PAGE or BN‐PAGE. Porin was probed as a housekeeping gene at 29 kDa. 5% = 5% of protein loaded into all other lanes. Each stage indicates a point in the carrier import process [29, 55, 61, 62].

Next, we assessed the requirement of a membrane potential and ATP hydrolysis for successful import and assembly of SFXN3 into mitochondria. To do so, SFXN3 was imported into wild‐type yeast *S. cerevisiae* mitochondria in the presence or absence of a membrane potential and ATP. Our data in Fig. 2A show that, similar to AAC, SFXN3 import strictly requires the membrane potential. ATP is known to facilitate the import of carrier proteins into yeast mitochondria by affecting the stage of receptor recognition (involving mainly Tom70) on the surface of mitochondria [26], and so all of our subsequent import assays were in the presence of ATP. SFXN3 behaves like a well‐known substrate for the carrier pathway. Importantly, BN‐PAGE data highlight the importance of the combination of ATP and membrane potential in the final assembly and insertion of SFXN3 into the inner membrane of yeast mitochondria. This final step in the import pathway of carrier proteins where the protein is fully inserted and assembled in the IM has been described as Stage V of the carrier import pathway [29]. Once this was determined, we investigated the degree to which SFXN3 depended on a membrane potential for its import and assembly compared to other carrier proteins such as AAC. To do so, we performed sequential incubations of SFXN3 with mitochondria under different conditions (Fig. 2B). In the first incubation, the absence of a membrane potential arrested the import of AAC and SFXN3 at a stage where it is inserted into the general TOM entry gate and accumulated in the *trans* side of the TOM complex, in interaction with the small Tim chaperones [26, 30], but not yet engaged with the IM insertion machinery. This has been described as Stage III of the carrier import pathway [29]. In the second incubation, following reestablishment of the membrane potential, both SFXN3 and AAC completed their insertion into the inner membrane. However, we noticed that in the BN‐PAGE analysis SFXN3 was less efficient in reaching Stage V of complete assembly into the inner membrane compared to AAC. This suggests that SFXN3 may require a higher membrane potential for complete assembly than AAC or alternatively, may require additional insertion components that rely on a membrane potential. Altogether, these findings unequivocally demonstrate that SFXN3 localises to the inner mitochondrial membrane and follows the carrier import pathway for entry into the mitochondria and insertion into the mitochondrial inner membrane. SFXN3, however, does not belong to the metabolite carrier family of proteins that all share a 6 transmembrane domain structure, and it is predicted to have either 4 or 5 transmembrane regions. This could suggest differences in its import and assembly compared to carrier proteins. For example, it may differ in terms of its interaction with small Tim chaperones or show a variable dependence on the inner membrane potential. These subtle variations from the classical carrier pathway may explain the difference in the formation of the final Stage V assembly species (Fig. 2B). Additionally, SFXN3 lacks the carrier signature motif, and its topology is likely determined in a different manner compared to carrier proteins in the inner membrane. Further dedicated studies are required to address this point in detail and to determine how different regions of the protein contribute to its import and insertion. Overall, these data provide us with a better understanding of the physiological, subcellular localisation of SFXN3 and the conditions required for its successful import into mitochondria. Determining the localisation and biochemistry of SFXN3 is a critical step towards improving our understanding of SFXN3’s molecular and cellular functions, as we proceed to do with the subsequent data.

### Proteomics analysis identifies expression level changes in neurodegeneration‐associated proteins downstream of SFXN3

**Fig. 3:**
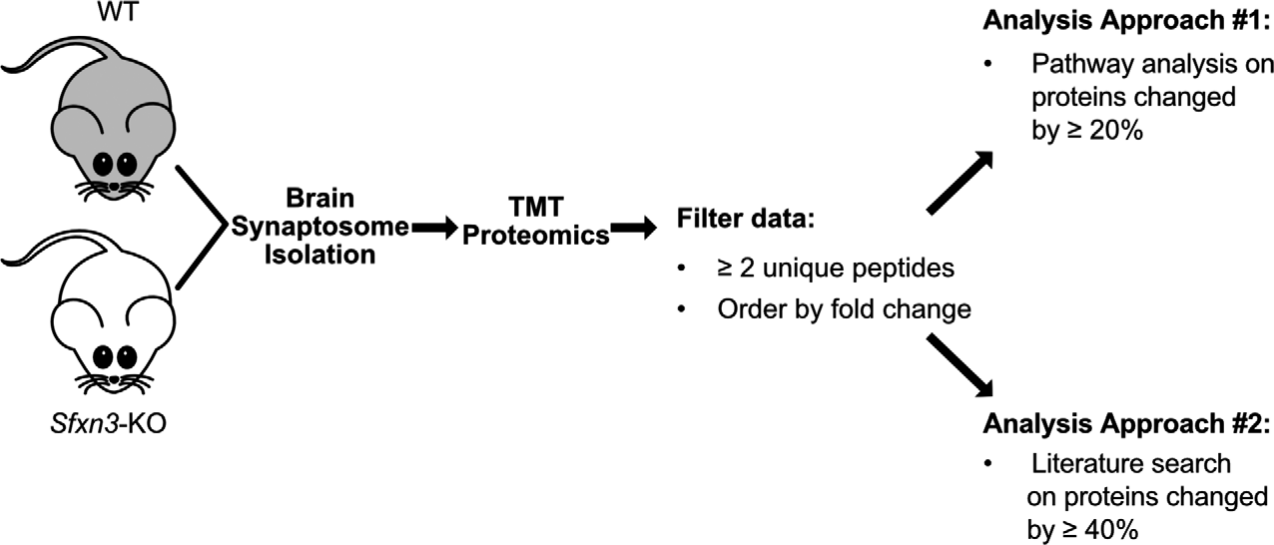
Proteomics experimental design schematic. Visual representation of steps to perform two separate analyses on proteomics data from WT and *Sfxn3‐*KO mice brain synaptosomes. TMT, Tandem Mass Tag. Fold change = *Sfxn3‐*KO/WT.

**Fig. 4:**
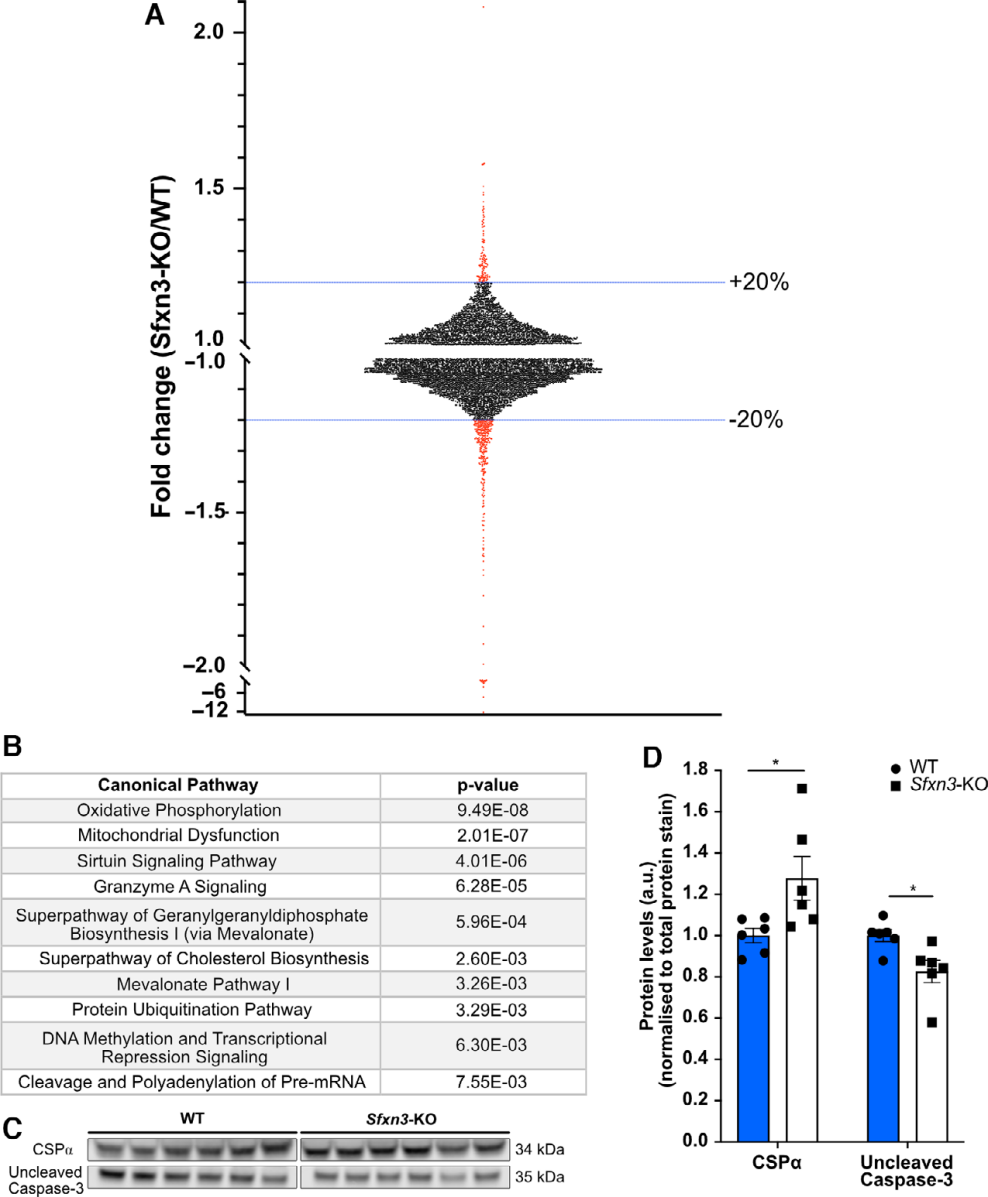
Proteomics analysis and immunoblot validation of *Sfxn3*‐KO synaptosomes. (A) Scatterplot of the fold change (*Sfxn3*‐KO/WT) distribution of 6,186 proteins identified by ≥ 2 unique peptides in proteomics screen of 1‐year‐old WT and *Sfxn3*‐KO mice brain synaptosomes. Dotted blue lines show the 20% up‐ and downregulated threshold and the specific data points beyond the threshold are indicated in red. (B) Top 10 canonical pathways identified from proteins altered by more than 20% in *Sfxn3*‐KO mice, as identified by Ingenuity Pathway Analysis. For complete proteomics dataset, see DataShare file: https://doi.org/10.7488/ds/3068. (C) Immunoblot protein bands for CSPα and Uncleaved Caspase‐3 in synaptosome isolates from 1‐year‐old WT and *Sfxn3*‐KO mice brains. (D) Quantification of CSPα (*P* = 0.026) and Uncleaved Caspase‐3 (*P* = 0.017) protein levels in synaptosomes of 1‐year‐old WT and *Sfxn3*‐KO mice brains. *P < 0.05 in Mann‐Whitney *U* (CSPα) and in two‐tailed unpaired *t*‐test (Uncleaved Caspase‐3). Quantitative data are shown as mean ± SEM (*N* = 6 mice per genotype). Data values from two technical replicates were combined to produce the resulting mean. See also Fig. 3 for proteomics experimental design scheme.

Once we confirmed the submitochondrial localisation of SFXN3 in human cells, and the conditions it requires for mitochondrial import, we wanted to identify proteins downstream of SFXN3 in neurons. To do so, we focused on the synaptosomal fractions of mouse brains due to the importance of synapses in neurodegenerative diseases and due to SFXN3 levels being 38% higher in synaptic versus non‐synaptic mitochondria [31]. Specifically, we performed 2‐way analysis with tandem mass tag (TMT) mass spectrometry on synaptosomes enriched from 1‐year‐old wild‐type (WT) and Sideroflexin 3 knockout (*Sfxn3*‐KO) mice brains (three female and three male mice per genotype). Data were pre‐filtered by proteins that had been identified by ≥ 2 unique peptides to ensure only reliable identifications were considered for further analysis (Fig. 3); this resulted in a total of 6186 proteins (see DataShare file: https://doi.org/10.7488/ds/3068). As depicted in Fig. 4A, of the 6186 proteins identified, most proteins identified in WT and *Sfxn3*‐KO synaptosomes did not differ substantially in terms of fold change. However, as indicated by the proteins in red which are up‐ or downregulated by more than 20%, a select group of proteins showed abundance levels that differed significantly between WT and *Sfxn3*‐KO synaptosomes. This suggested that SFXN3 is not a master regulator, but rather is involved in changing expression levels of key target proteins.

**Fig. 5:**
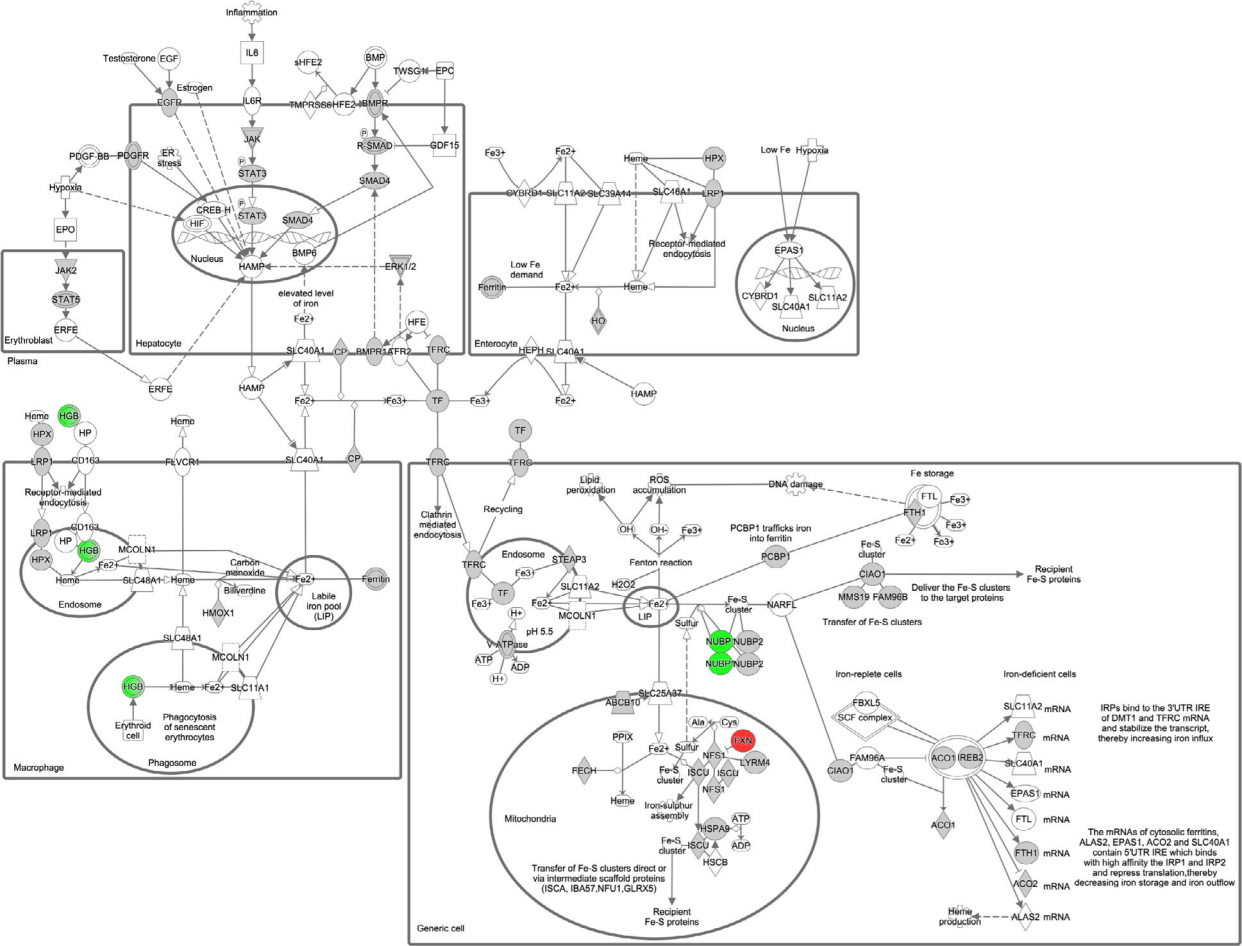
Iron homeostasis signalling pathway showing proteins identified by ≥ 2 unique peptides and fold change ≥ 20%, mapped by IPA. Green nodes represent downregulated proteins, red nodes represent upregulated proteins, grey nodes represent proteins that were identified in the proteomics dataset but whose expression levels were not significantly altered by a fold change ≥ 20%, and white nodes represent proteins or groups/families of molecules that were either not present or sufficiently represented (respectively) in the proteomics dataset. Solid lines represent direct interactions and dotted lines represent indirect interactions. Double circle represents a family of proteins. FXN, Frataxin; HGB, Haemoglobin; NUBP1, nucleotide binding protein 1.

As SFXN3 belongs to a family of putative iron transporters [12], we were curious about the effect of SFXN3 loss on iron homeostasis and iron synthesis‐related proteins. Therefore, we aligned our proteins to ingenuity pathway analysis (IPA)‐derived lists of molecules involved in iron homeostasis and iron synthesis as well as proteins involved in the iron homeostasis signalling pathway. Here we identified 3 proteins that showed a fold change ≥ 20% relative to controls (see DataShare file: https://doi.org/10.7488/ds/3068 and Fig. 5). These included the adult beta chain of haemoglobin, Hbb‐b1, and iron‐sulfur cluster biogenesis‐related proteins NUBP1 and Frataxin. Specifically, Hbb‐b1 and cytosolic protein NUBP1 were downregulated with a fold change (*Sfxn3*‐KO/WT) of −1.37, −1.61, respectively, and mitochondrial protein Frataxin was upregulated with a fold change of 1.35 [32, 33]. The identification of these iron‐related proteins is particularly interesting since iron dysregulation has been found to be implicated in neurodegenerative diseases, and more specifically, dopaminergic neurodegeneration [34, 35, 36]. In the case of Hbb‐b1, Biagioli et al. [37] found that this iron‐related protein plays a role in the function of dopaminergic neurons in the substantia nigra by modulating iron metabolism, oxygen homeostasis, and oxidative phosphorylation. Furthermore, NUBP1 and Frataxin are iron‐sulfur cluster biogenesis‐related proteins which play a crucial role in various biological processes, including mitochondrial respiration, protein translation, and DNA repair [38]. In fact, mutations and dysregulated biogenesis of iron‐sulfur cluster proteins can lead to metabolic, haematologic and neurological diseases, such as Friedreich’s ataxia and PD [39, 40]. Of importance to consider in the interpretation of this data is the study by Amorim et al. [10], which did not identify perturbations in the mitochondrial respiratory chain of 3‐month‐old *Sfxn3*‐KO mice mitochondria. As iron ions are essential components of respiratory chain complexes, the extent to which changes in these iron‐related protein levels are functionally relevant remains unclear but warrants further investigation.

To further identify functionally relevant proteins and pathways affected by SFXN3, two analysis approaches were adopted (Fig. 3). The first approach involved submitting the pre‐filtered 6186 proteins to IPA and using a cut‐off of 20% change relative to controls to perform bioinformatic analyses. Furthermore, this approach highlighted the top 10 canonical pathways predicted to be affected in proteins showing a ≥ 20% change (Fig. 4B). The top canonical pathways as ranked by p‐value (i.e. overlap with canonical protein lists) included oxidative phosphorylation and mitochondrial dysfunction with the smallest *P*‐values, meaning the data were most enriched for proteins that belonged to those canonical pathways. Of interest within the mitochondrial dysfunction canonical pathway was the downregulation of a single protein, uncleaved Caspase‐3 with a fold change (*Sfxn3*‐KO/WT) of −1.30. Since SFXN3 is a mitochondrial protein, it is not particularly surprising that these pathways appear to be the most enriched within the dataset. However, it is important to note that an enrichment in these pathways does not signify that oxidative phosphorylation (OXPHOS) complexes are disrupted, as supported by data in Amorim et al. [10], or that mitochondrial dysfunction is present in *Sfxn3*‐KO synaptosomes. Instead, it indicates that there are changes in the proteins associated with these pathways.

The second approach involved a literature search on proteins with a 40% change relative to controls, a cut‐off which resulted in a total of 62 proteins. This enabled a more target‐driven approach to identify potential mediators downstream of SFXN3 at the synapse. Proteins with a 40% change relative to controls were searched on PubMed along with the terms neurodegeneration/neurodegenerative, synapse/synaptic and Parkinson. This highlighted CSPα as a protein of particular interest with a fold change (*Sfxn3*‐KO/WT) of 1.51. Based on their significant fold change relative to controls and their association with cell death and neurodegeneration, uncleaved Caspase‐3 and CSPα were both selected for further analysis [41, 42, 43]. The proteomics results were validated by immunoblotting for uncleaved Caspase‐3 and CSPα in synaptosomes from 1‐year‐old mice and results showed a significant increase in CSPα levels by 28% and a significant decrease in uncleaved Caspase‐3 levels by 17.5% (Fig. 4C,D). The identification of CSPα and uncleaved Caspase‐3 as downstream target proteins of SFXN3 suggests a role for SFXN3 in the modulation of cell death and neurodegenerative processes.

### Overexpression of an Sfxn3 orthologue rescues neuronal death in *Drosophila* models of Parkinsonian disease

**Fig. 6:**
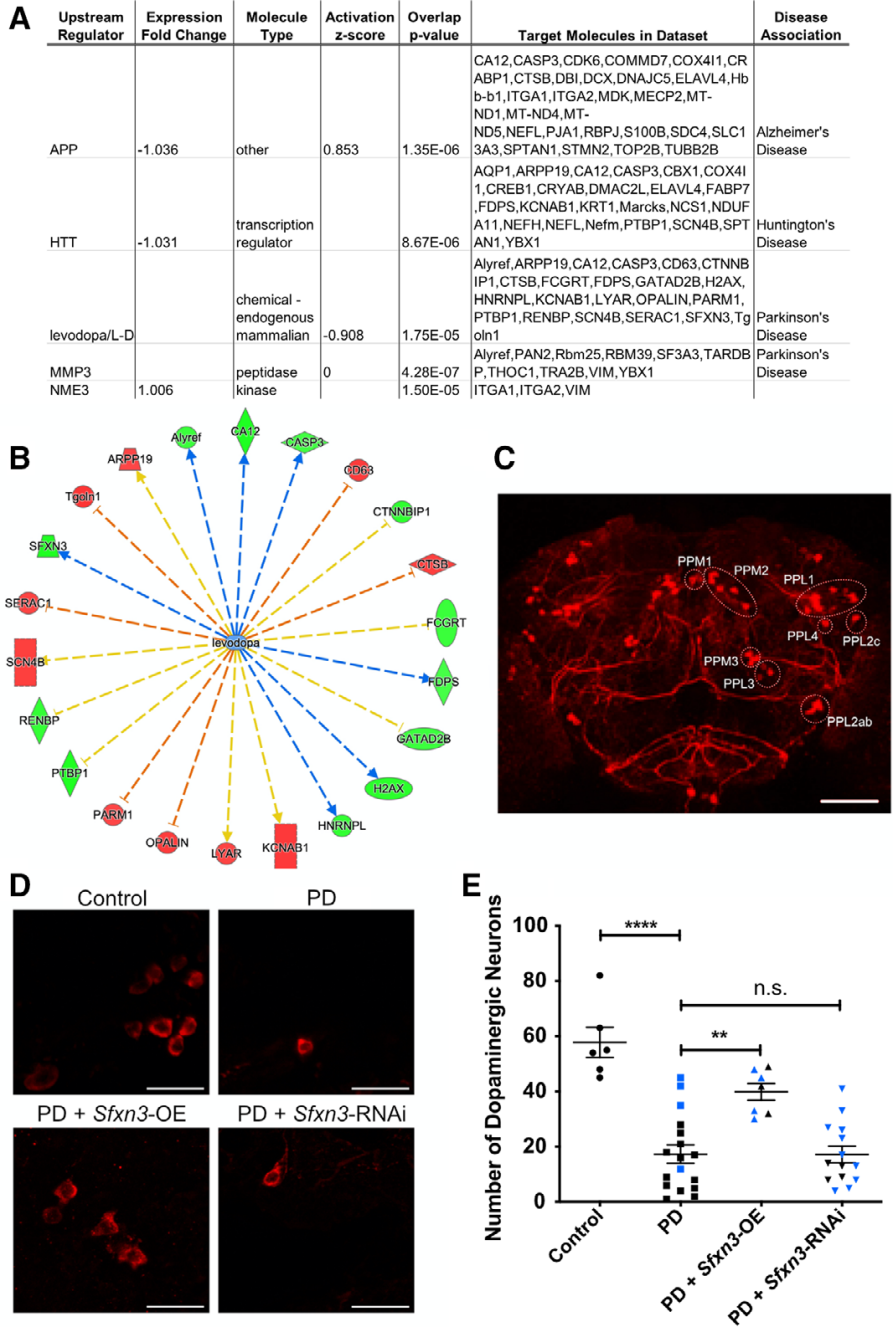
Sfxn3 orthologue overexpression ameliorates dopaminergic neuron loss in *Drosophila* models of Parkinson's disease. (A) Bioinformatic analysis of predicted upstream regulators of SFXN3, based on our proteomic data, revealed a significant association with pathways associated with Parkinson's disease, Alzheimer’s disease and Huntington’s disease. APP, Amyloid beta precursor protein; HTT, Huntingtin; MMP3, Stromelysin‐1; NME3, Nucleoside diphosphate kinase 3. Using molecule nomenclature as it appears in IPA. (B) Visual representation of the SFXN3‐dependent protein alterations predicted known to be associated with L‐Dopa/levodopa (red nodes = proteins with increased expression; green nodes = proteins with decreased expression in our proteomic dataset; orange arrows = leads to inhibition; blue arrows = leads to activation; yellow arrows = findings inconsistent with state of downstream molecule). CASP3 = Caspase‐3; CD63 = CD63 antigen; CTNNBIP1 = Beta‐catenin‐interacting protein 1; CTSB = Cathepsin B; FCGRT = IgG receptor FcRn large subunit p51; FDPS = Farnesyl pyrophosphate synthase; GATAD2B = Transcriptional repressor p66‐beta; H2AX = Histone H2AX; HNRNPL = Heterogeneous nuclear ribonucleoprotein L; KCNAB1 = Voltage‐gated potassium channel subunit beta‐1; LYAR = Cell growth‐regulating nucleolar protein; PARM1 = Prostate androgen‐regulated mucin‐like protein 1; PTBP1 = Polypyrimidine tract‐binding protein 1; RENBP = N‐acylglucosamine 2‐epimerase; SCN4B = Sodium channel subunit beta‐4; SFXN3 = Sideroflexin 3; Tgoln1 = Trans‐Golgi network integral membrane protein 1; ARPP19 = cAMP‐regulated phosphoprotein 19; Alyref = THO complex subunit 4; CA12 = Carbonic anhydrase 12. Using molecule nomenclature as appears in IPA. (C) Representative low‐power confocal micrograph of dopaminergic neurons in a control *Drosophila* brain, revealing the anatomical location of distinct neuronal clusters (labelled on the right side of the micrograph following the nomenclature in Mao and Davis [63]). Scale bar: 50 μm. (D) Representative high‐power confocal micrographs of dopaminergic neurons in control, PD, PD + *Sfxn3*‐OE and PD + *Sfxn3*‐RNAi *Drosophila* brains immunostained for tyrosine hydroxylase. Scale bar: 10 μm. (E) Quantification of dopaminergic neurons in control, PD, PD + *Sfxn3*‐OE and PD + *Sfxn3*‐RNAi *Drosophila* brains. Data points are colour coded to differentiate between the two PD models. In the quantification of PD, PD + *Sfxn3*‐OE and PD + *Sfxn3*‐RNAi *Drosophila* brains, data points in blue correspond to *Drosophila* expressing mutant α‐synuclein (A53T) and data points in black correspond to *Drosophila* expressing mutant α‐synuclein (A30P). Each point represents one *Drosophila* brain, n.s. = *P* > 0.05, **P < 0.01, ****P < 0.0001 in one‐way ANOVA with a Tukey’s multiple comparison test. Graphs plotted as mean ± SEM.

As the proteomics analysis highlighted that SFXN3 associates with key neurodegeneration proteins including CSPα and uncleaved Caspase‐3, we hypothesised that the manipulation of SFXN3 levels may modulate neurodegeneration‐associated cascades in neurodegenerative diseases. To explore this possibility further, we performed a predicted upstream regulator analysis of our proteomic data using IPA software. These analyses confirmed that four of the top five predicted upstream regulators (identified by IPA based on p‐value) of the proteomic differences observed in *Sfxn3*‐KO synapses are known regulators/causes of neurodegenerative disease (Fig. 6A): L‐Dopa/levodopa and MMP3 (Parkinson's disease), APP (Alzheimer’s disease), and HTT (Huntington’s disease). Thus, many of the downstream consequences of SFXN3 include the altered expression of proteins previously associated with neurodegenerative disease.

Interestingly, several previous studies include experimental data suggesting dysregulation of SFXN3 mRNA and/or protein levels in animal models and patients with PD [14, 15, 16]. Taken together with our novel proteomics findings, this suggested that further investigation of the potential role of SFXN3 in PD‐associated neurodegeneration was warranted. Additional bioinformatic interrogation of our newly identified L‐Dopa/PD pathway revealed that SFXN3 knockdown alters the expression of 22 individual proteins associated with PD‐dependent pathways (Fig. 6B). To test the possibility that manipulation of SFXN3 may be sufficient to modulate PD‐dependent neurodegenerative pathways *in vivo*, we asked whether experimental manipulation of an *Sfxn3* orthologue (see Materials & Methods) could modify neurodegenerative phenotypes in a *Drosophila* model of PD. We crossed elav‐Gal4 driver strains with lines harbouring an overexpression or knock‐down of an *Sfxn3* orthologue under the upstream activating sequence (UAS) enhancer sequence in *Drosophila* models of PD expressing mutant α‐synuclein (A53T or A30P). Two *Drosophila* models of PD were examined because we were interested in the ability of *Sfxn3* to modulate neuronal stability in the context of PD, irrespective of the causative mutation. Specifically, since PD is characterised by the loss of dopaminergic neurons [8], we assessed the effect of modulating *Sfxn3* levels on the number of dopaminergic (i.e. tyrosine hydroxylase positive) neurons in the *Drosophila* brain (Fig. 6C). Firstly, as expected, we observed a significant downregulation in the number of dopaminergic neurons in PD fly models compared to control flies (Control: mean ± SEM = 57.83 ± 5.46 vs. PD: mean ± SEM = 17.29 ± 3.33) (Fig. 6D,E). The extent of dopaminergic neuron loss observed in the combined counts of A53T and A30P expressing PD fly models is consistent with previous findings in Mohite et al. and Botella, Bayersdorfer and Schneuwly when considering that we investigated *Drosophila* brains at a later time point (7‐weeks‐old) [44, 45]. Importantly, we observed that *Sfxn3* overexpression in the PD fly models significantly rescued the reduction in dopaminergic neurons seen in the combined A53T or A30P counts (PD: mean ± SEM = 17.29 ± 3.33 vs. PD + *Sfxn3*‐OE: mean ± SEM = 39.86 ± 3.03) (Fig. 6D,E). In contrast, downregulation of *Sfxn3* in PD flies via *Sfxn3*‐RNAi had no effect on the loss of dopaminergic neurons (PD: mean ± SEM = 17.29 ± 3.33 vs. PD + *Sfxn3*‐RNAi: mean ± SEM = 17.21 ± 2.99). Thus, increased expression of an *Sfxn3* orthologue in *Drosophila* was sufficient to ameliorate neurodegenerative changes *in* *vivo* triggered by disease‐causing genetic mutations. It is likely that the aforementioned interaction between SFXN3 and neurodegeneration‐related proteins such as uncleaved Caspase‐3 and CSPα enables *Sfxn3* overexpression to modulate these protein levels in a manner that protects dopaminergic neuron degeneration. These data demonstrate that *Sfxn3* overexpression is neuroprotective in the context of dopaminergic neuron loss in PD fly models.

### Absence of SFXN3 does not trigger neurodegeneration *in vivo*

**Fig. 7:**
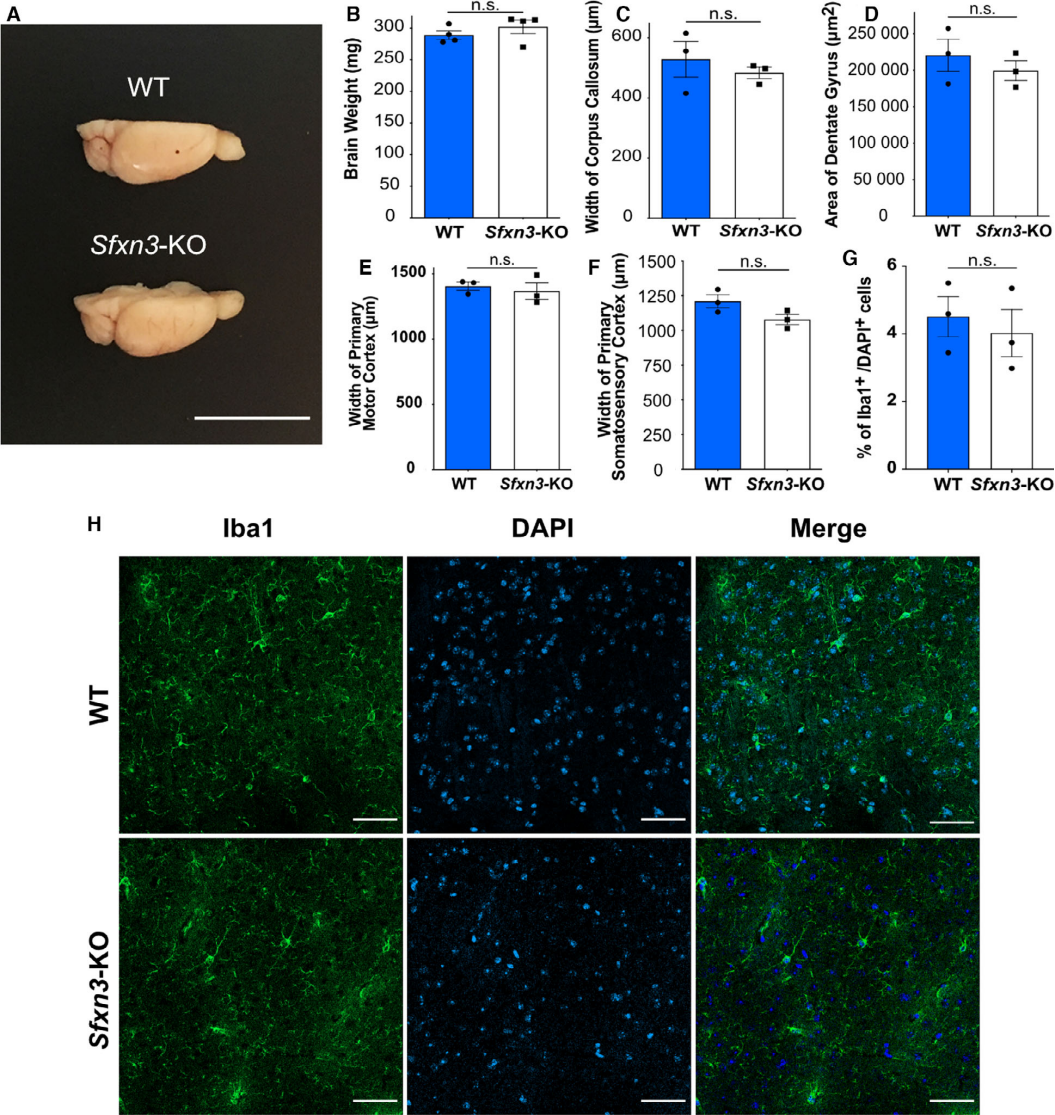
Phenotypic characterisation of 1‐year‐old *Sfxn3*‐KO mice. (A) Representative image of the right hemisphere of 1‐year old WT and *Sfxn3*‐KO mouse brains. Scale bar = 1 cm. (B–F) Quantification of weight of the brain’s right hemisphere (*P* = 0.47), width of corpus callosum (*P* = 0.51), area of dentate gyrus (*P* = 0.46), width of primary motor cortex (*P* = 0.62) and width of primary somatosensory cortex (*P* = 0.09) of 1‐year old WT and *Sfxn3*‐KO mice. (B) *N* = 4, n.s. = *P* > 0.05 in two‐tailed unpaired t‐test. (C, E, F) *N* = 3, *n* = 27, n.s. = *P* > 0.05 in two‐tailed unpaired t‐test. (D) *N* = 3, *n* = 9, n.s. = *P* > 0.05 in two‐tailed unpaired t‐test. (G) Quantification of microglia (Iba1^+^ cells) as a percentage of Iba1^+^/DAPI^+^ cells in order to normalise the number of Iba1^+^ to total cell number per area. *N* = 3 mice per group, *n* = 9 measurements per group, n.s. = *P* > 0.05 in two‐tailed unpaired t‐test and *P* = 0.73. (H) Confocal images of 1‐year‐old WT and *Sfxn3*‐KO brain sections where the dentate gyrus is first fully formed (Bregma −1.46 mm). Sections were stained for microglial marker, Iba1 and nuclear marker, DAPI. Scale bar: 50 μm. (B–G) Graphs plotted as mean ± SEM.

**Fig. 8:**
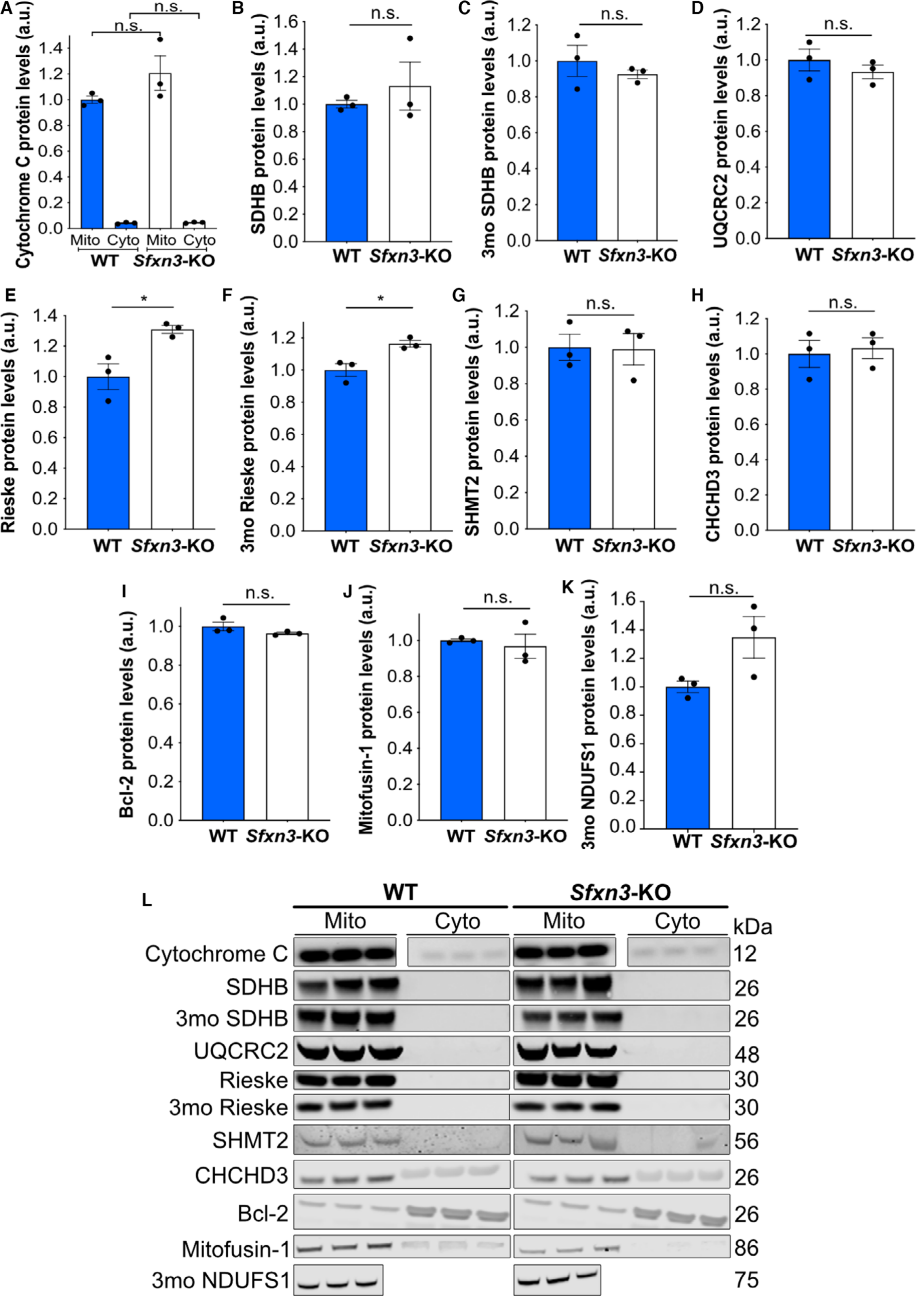
Molecular characterisation of 3‐month‐old and 1‐year‐old *Sfxn3*‐KO mice. (A, B, D, E, G–J) Quantification of Cytochrome C (mitochondria comparison had a *P* = 0.22 and cytoplasm comparison had a *P* > 0.99), OXPHOS Complex II/SDHB (*P* > 0.99), OXPHOS Complex III/UQCRC2(*P* = 0.40), Rieske (*P* = 0.02), SHMT2 (*P* = 0.93), CHCHD3 (*P* = 0.75), Bcl‐2 (*P* = 0.18) and Mitofusin‐1 (*P* = 0.70) in mitochondrial and cytoplasmic fractions of WT and *Sfxn3*‐KO mouse brains at 1‐year of age. (C, F, K) Quantification of OXPHOS Complex II/SDHB (*P* = 0.45) and Rieske (*P* = 0.02) in mitochondrial and cytoplasmic brain fractions and NDUFS1 (*P* = 0.08) in brain protein lysate from WT and *Sfxn3*‐KO mice at 3‐months‐old (3mo). (A) *N* = 3, n.s. = *P* > 0.05 in one‐way ANOVA with a Tukey’s multiple comparison test. (B, J) *N* = 3, n.s. = *P* > 0.05 in Mann‐Whitney *U* test. (C–I, K) *N* = 3, n.s. = *P* > 0.05 and *P < 0.05 in two‐tailed unpaired *t*‐test. (A–K) Graphs plotted as mean ± SEM. (L) Immunoblot protein bands for Cytochrome C, OXPHOS Complex II/SDHB, OXPHOS Complex III/UQCRC2, Rieske, SHMT2, CHCHD3, Bcl‐2, Mitofusin‐1 in mitochondrial and cytoplasmic fractions of WT and *Sfxn3*‐KO mouse brains at 1‐year of age. Immunoblot protein bands for OXPHOS Complex II/SDHB and Rieske in mitochondrial and cytoplasmic brain fractions and NDUFS1 in brain protein lysate in WT and *Sfxn3*‐KO mice at 3‐months‐old (3mo) are also shown. Respective molecular weights in kDa are shown on the right‐hand side of the panel.

The manipulation of *Sfxn3* levels in *Drosophila* models of PD showed that *Sfxn3* overexpression rescued dopaminergic neuron loss in contrast to *Sfxn3* downregulation, which did not induce any further neurodegeneration. To confirm our findings that show *Sfxn3* downregulation is not sufficient to trigger neurodegeneration, we used a mammalian model system lacking *Sfxn3*. Specifically, we performed experiments using aged (1‐year‐old) *Sfxn3*‐KO mice to examine their role in the long‐term stability of the nervous system. First, we compared the size and weight of 1‐year‐old WT and *Sfxn3*‐KO mice brains and observed no differences between genotypes (Fig. 7A,B). We also performed detailed morphological analyses comparing different grey and white matter brain areas. Interestingly, we found no differences between 1‐year‐old *Sfxn3*‐KO and WT mice when comparing the width of the corpus callosum, the area of the dentate gyrus, the width of the primary motor cortex and the width of the primary somatosensory cortex (Fig. 7C–F). Next, we wanted to investigate whether there was any inflammation in the brains of *Sfxn3*‐KO mice since inflammation plays an important role in neurodegeneration across several diseases [46]. To do so, brain sections were immunostained for Iba1, to probe for activated microglia (Fig. 7H). Following quantification, we found no difference in the percentage of microglia in WT versus *Sfxn3*‐KO brains (Fig. 7G). Similarly, immunoblots for proteins associated with mitochondrial health [e.g. Cytochrome C, OXPHOS Complex II (SDHB), OXPHOS Complex III (UQCRC2)] and mitochondrial integrity (e.g. CHCHD3, Bcl‐2 and Mitofusin‐1) revealed no differences in their levels in *Sfxn3*‐KO relative to controls (Fig. 8A,B,D,H–J,L). Thus, our mammalian model system confirmed that the loss of SFXN3 was not sufficient to trigger any overt neurodegeneration *in vivo*, even with increasing age, suggesting that SFXN3 plays a role in modifying/delaying, rather than triggering, neurodegeneration. The lack of an overt neurodegenerative phenotype in these *Sfxn3*‐KO mice is comparable to Wallerian degeneration slow (Wld^S^) mutant mice, which similarly, do not exhibit an overt phenotype unless challenged with injury or disease [47]. It is in this scenario that basal alterations in mitochondrial proteins may then provide a neuroprotective response to neurodegenerative stimuli.

### SFXN3 regulates levels of certain iron‐related proteins, but not the one‐carbon metabolism protein SHMT2

Given the identification of iron‐related proteins Hbb‐b1, Frataxin and NUBP1 downstream of SFXN3 in our proteomics dataset, we wanted to further investigate potential expression changes in other iron‐related proteins in our 1‐year‐old *Sfxn3*‐KO mice. As mentioned previously, since mitochondrial bioenergetics remain intact in 3‐month‐old *Sfxn3*‐KO mice, it puts into question the functional relevance of expression changes observed in the iron‐related proteins above [10]. However, as our mice were 1‐year‐old, we wanted to determine whether there were changes in iron‐related proteins over time which may suggest potential functional relevance. To do so, we assessed the levels of iron‐sulfur proteins NDUFS1, SDHB and Rieske, which are subunits of OXPHOS Complexes I, II and III, respectively, in 3‐month and 1‐year‐old mice. There was no statistically significant difference in the levels of SDHB between WT and *Sfxn3*‐KO mice at 3 months nor at 1 year of age (Fig. 8B,C,L). Furthermore, NDUFS1 protein levels were not significantly different between genotypes at 3‐months‐old but did appear to show an upwards trend in expression in *Sfxn3*‐KO mice (Fig. 8K,L). As for Rieske, it showed a statistically significant increase in *Sfxn3*‐KO mice relative to controls at both 3 months and 1 year of age (Fig. 8E,F,L). Since frameshift mutations in *Sfxn1* cause iron accumulation in mitochondria [12], this could also be the case in mitochondria of *Sfxn3*‐KO mice. If so, this might explain the increase in mitochondrial levels of the Rieske iron‐sulfur protein we see in *Sfxn3*‐KO mice. Interestingly, however, this potential mechanism for an increase in Rieske does not appear to affect levels of other iron‐sulfur cluster proteins such as NDUFS1 and SDHB to the same degree. This may be due to varying sensitivities to increased mitochondrial iron levels across distinct iron‐sulfur cluster proteins. Lastly, we assessed levels of the serine catabolic enzyme SHMT2, involved in one‐carbon metabolism [48]. As SFXN1 and SFXN3 are thought to play a role in the one‐carbon metabolism pathway [13], we wanted to investigate whether SFXN3 affects levels of SHMT2 *in vivo*. Interestingly, we found that *Sfxn3*‐KO had no effect on the protein levels of SHMT2 (Fig. 8G,L). Overall, these results show that the loss of *Sfxn3* can affect levels of iron‐sulfur cluster proteins such as the Rieske protein, but does not alter levels of SHMT2, the first rate‐limiting enzyme in the one‐carbon metabolism pathway [48].

## Conclusions

SFXN3 belongs to a family of highly related mitochondrial transmembrane proteins consisting of SFXN1–5 [11]. As is the case of SFXN3, there are sparse publications on other members of the SFXN family. However, the studies that are available highlight various possible roles across members of the SFXN family. For example, mutations in SFXN1 are known to cause siderocytic anaemia with hallmark mitochondrial iron accumulation [12], and Kory et al. [13] have also shown it plays a role as a serine transporter in one‐carbon metabolism. SFXN2 plays a role in mitochondrial iron metabolism and causes impaired mitochondrial respiration in *SFXN2*‐KO cells [49]. SFXN4 is required for iron‐sulfur cluster biogenesis, iron homeostasis, haem synthesis and mitochondrial respiration [50]. As mentioned previously, it is important to note that in contrast to SFXN2 and SFXN4, mitochondrial respiration is unaffected in *Sfxn3*‐KO mice [10]. Lastly, SFXN5, along with SFXN2 and SFXN3, is thought to play a role in the regeneration of pancreatic endocrine cells [24]. Based on these (and possibly other as yet unidentified) diverse functions of each member of the SFXN family, it is perhaps not surprising that the novel finding of SFXN3 as a modulator of neurodegeneration has not been associated with the other SFXN family members.

The mitochondrial protein SFXN3 was recently identified as a neuronally enriched protein with strong expression in synaptic terminals. In the present study, we provide further insights into the mitochondrial localisation and import of SFXN3. We demonstrate that SFXN3 regulates levels of proteins known to be associated with neurodegeneration and cell death pathways (including CSPα and Caspase‐3), as well as constituents of pathways directly associated with several neurodegenerative diseases. Altered expression of an *Sfxn3* orthologue in *Drosophila* models of PD ameliorated the loss of dopaminergic neurons. Taken together, these results demonstrate a role for SFXN3 in the mitochondrial axis of regulation of neurodegeneration pathways. Furthermore, they encourage further research into SFXN3 as a potential therapeutic target for modulating neurodegenerative cascades underlying neurodegenerative conditions.

## Materials and methods

### Data and code availability

The proteomics data generated during this study are publicly available on DataShare at https://doi.org/10.7488/ds/3068. This paper does not report original code. Any additional information required to reanalyse the data reported in this paper is available from the lead contact upon request.

### Animals

Sfxn3^tm1b(KOMP)Wtsi^ mice (*Sfxn3*‐KO mice; http://www.mousephenotype.org/data/genes/MGI:2137679; RRID: IMSR_EM:07902) were obtained from the Wellcome Trust Sanger Institute Mouse Genetics Project as part of the nPad MRC Mouse Consortium, and maintained on a C57Bl/6N background. Wild‐type C57Bl/6NTac mice, obtained from MRC Harwell, were used as experimental controls. Mice used were of mixed gender and were sacrificed at either 3 months or 1 year of age. All mice were housed within the animal care facilities at the University of Edinburgh under standard SPF conditions in conventional open‐top cages. All animals were bred and handled following the UK Animals (Scientific Procedures) Act, 1986. Procedures were approved by the internal ethics committee at the University of Edinburgh and follow UK Home Office regulations.

### Cell culture

Human embryonic kidney (HEK) 293 cells were cultivated under standard conditions (37 °C, 5% CO_2_ atmosphere) in Dulbecco’s modified Eagle medium (DMEM) (Thermo Fisher Scientific, Waltham, MA, USA, Cat #: 41965‐039), supplemented with 10% (v/v) fetal bovine serum (FBS) (Thermo Fisher Scientific, 10500‐064), and antibiotics (100 units penicillin and 100 μg streptomycin per mL (Sigma‐Aldrich, St. Louis, MO, USA, Cat #: P0781)).

### Isolation of mouse brain and HEK293 mitochondria

Mitochondrial isolation was performed via differential centrifugation as previously described [51]. Mouse forebrain tissue was homogenised in mitochondria isolation buffer (MIB) (210 mm Mannitol, 70 mm Sucrose, 5 mm HEPES, 1 mm EGTA, 0.5% BSA, 1% Protease Inhibitor Cocktail (Roche, Basel, Switzerland, Cat # 11836153001), pH 7.2) using a glass mortar and pestle, manually, on ice, and the resulting homogenate was centrifuged (1000 ***g***, 5 min, 4 °C). The supernatant was then collected and centrifuged (12 000 ***g***, 10 min, 4 °C) and this step was performed twice to acquire both the mitochondrial and cytoplasmic fractions. The mitochondrial pellet was washed in 1 mL MIB, centrifuged (12 000 ***g***, 10min, 4 °C) and finally resuspended in minimal volume of MIB. Protein concentration was measured using the Micro BCA Assay (Thermo Fisher Scientific). For HEK293 mitochondria isolation, 5 x T‐75 flasks were used. Cells were scraped off in their medium, on ice, and centrifuged (1000 ***g***, 5 min, 4 °C). The pellet was resuspended in 5 mL ice‐cold PBS and centrifuged again. The pellet was then treated the same way as the forebrain tissue, with homogenisation in MIB as the following step.

### Sodium carbonate extraction

We followed the basic procedure published by Fujiki et al. [18] with some minor modifications. Specifically, 100 μg mitochondria were extracted from HEK293 cells, as described above, and incubated in 100 μL of 100 mm freshly made sodium carbonate (Na_2_CO_3_) pH 11.5 for 20 min on ice. The membranes were collected by ultracentrifugation at 100 000 ***g*** for 30 min, 4 °C. The pellet was resuspended in 100 μL sodium carbonate and both supernatant (soluble fraction) and pellet (insoluble fraction) were subjected to trichloracetic acid (TCA) precipitation. More specifically, addition of 1 μL 1.25% Na‐deoxycolate and 25 μL of 72% TCA was followed by a 30 min incubation on ice. The samples were then centrifuged at 14 000 ***g*** for 30 min, 4 °C. The pellets were washed with 400 μL ice‐cold acetone and centrifuged for another 10 min at 14 000 ***g***, 4 °C. Finally, the samples were analysed by SDS‐PAGE and immunoblotting as described in “Immunoblotting of HEK293 and yeast proteins” using the following antibodies: Mitofusin 2 (Proteintech, Rosemont, IL, USA, Cat #: 12186‐1‐AP, 1 : 1000), SFXN3 (Atlas Antibodies, Bromma, Stockholm, Sweden, Cat #:HPA008028, 1 : 500), TFAM (Abnova, Taipei, Taiwan, Cat #: H00007019‐B01P, 1 : 1000), Goat Anti‐Rabbit IgG (whole molecule)–Peroxidase secondary antibody (Sigma‐Aldrich, Cat #: A6154, 1 : 10 000) and Goat Anti‐Mouse IgG, H&L Chain Specific–Peroxidase secondary antibody (Sigma‐Aldrich Cat #: 401215, 1 : 10 000).

### Swelling and proteinase K protection assay

100 μg of mitochondria were freshly isolated from HEK293 cells, as described above, and resuspended in 200 μL of either isotonic buffer (250 mm sucrose, 1 mm EDTA, 10 mm Tris/HCl pH 7.6) or hypotonic buffer (1 mm EDTA, 10 mm Tris/HCl pH 7.6) on ice. Briefly, a 45 min incubation was followed by centrifugation (12 000 ***g***, 5 min, 4 °C) to pellet the resuspended mitochondria. In one sample per treatment 50 μg·mL^−1^ proteinase K was added in 100 μL isotonic buffer for 30 min on ice and was inactivated by the addition of 2 mm PMSF for 10 min. Mitochondria were pelleted by centrifugation (12 000 ***g***, 5 min, 4 °C) and all samples were analysed by SDS‐PAGE followed by immunoblotting as described in “Immunoblotting of HEK293 and yeast proteins.” The antibodies used were the following: Mitofusin 2 (Proteintech, Rosemont, IL, USA, Cat #: 12186‐1‐AP, 1 : 1000), Mitofilin (Proteintech Cat #: 10179‐1‐AP, 1 : 1000), CHCHD3 (Proteintech Cat #: 25625‐1‐AP, 1 : 2000), SFXN3 (Atlas Antibodies Cat #: HPA008028, 1 : 500), TFAM (Abnova Cat #: H00007019‐B01P, 1 : 1000), Goat Anti‐Rabbit IgG (whole molecule)–Peroxidase secondary antibody (Sigma‐Aldrich Cat #: A6154, 1 : 10 000) and Goat Anti‐Mouse IgG, H&L Chain Specific–Peroxidase secondary antibody (Sigma‐Aldrich Cat #: 401215, 1 : 10 000).

### Immunoblotting of HEK293 and yeast proteins

For SDS‐PAGE, mitochondria were mixed with Laemmli sample buffer (10% SDS, 50% Glycerol, 60 mm Tris/HCl pH 6.8, 100 mm DTT, 0.1% Bromophenol Blue, 5% β‐mercaptoethanol), heated for 5 min at 95 °C, and separated by SDS‐PAGE (12% gel, 1% SDS) run at 150 V for 2 h at room temperature. Proteins were transferred to a 0.45‐μm nitrocellulose membrane (Thermo Fisher Scientific) using Semi‐dry Electroblotting (Transfer‐Blot SD, Bio‐Rad, Hercules, CA, USA) at 25 V for 25 min at room temperature. For BN‐PAGE, following the import protocol and pelleting of mitochondria via centrifugation detailed in “Import into yeast mitochondria,” mitochondria were resuspended in solubilisation buffer containing 1% digitonin and incubated on ice for 5 mins. Solubilised mitochondria were then centrifuged at 18 000 ***g*** at 4 °C for 30 min and the supernatant was transferred to a clean tube. To the supernatant, 5–10 μL of Sample Buffer (750 mm aminocaproic acid, 50 mm Bis–Tris/HCl, pH 7.0, 0.5 mm EDTA, and 5% Serva Blue G) was added, and the prepared sample was loaded onto a 5–13% gradient gel. The gel was run at 90 V for 30 min and then at 12 mA until the dye front reached the end of the gel. Proteins were then transferred to a PVDF membrane (Thermo Fisher Scientific) using Semi‐dry Electroblotting (Transfer‐Blot SD, Bio‐Rad) at 25 V for 25 min at room temperature.

In the case of radiolabelled proteins, the nitrocellulose or PVDF membranes were left in autoradiography cassettes with autoradiography screens for 48 h to allow the signal to develop. Radiolabelled proteins were then visualised by digital autoradiography (Molecular Dynamics, Sunnyvale, CA, USA) using the X‐OMAT developer. Instead, for the visualisation of non‐radiolabelled proteins, nitrocellulose membranes were blocked with 5% semi‐skimmed milk in TBS/Tween‐20 for 1 h at room temperature. Membranes were then incubated with primary antibodies overnight at 4 °C. Membranes were washed 5 times for 10 min each time with TBS/Tween‐20 and incubated with secondary antibody for 1 h at room temperature. Membranes were then washed 5 times for 10 min with TBS/Tween‐20 protein bands were visualised using electrochemiluminescence (Thermo Fisher Scientific) and X‐ray film (Thermo Fisher Scientific) with an automatic film processor.

### Isolation of yeast mitochondria

Wild‐type mitochondria, Tom70‐depleted (ΔTom70) mitochondria (YSC3869‐202335173; Horizon Discovery, Waterbeach, England, UK), Tim9 temperature sensitive (Tim9ts) [30] and Tim22 temperature sensitive (Tim22ts) knocked down mitochondria [52] were isolated from the *Saccharomyces cerevisiae* strain BY4741 (MATa his3Δ1 leu2Δ0 met15Δ0 ura3Δ0) and from the ΔTom70, Tim9ts, Tim22ts strains, as described previously. Briefly, the yeast strains were grown at 30 °C in lactate medium containing 1% (wt/vol) yeast extract, 2% (wt/vol) bacto‐peptone, and 2% lactate (vol/vol), pH 5.5 (YPL) to an OD_600_ of 1. Cells were harvested by centrifugation at 2500 ***g*** and then incubated in 0.1 m Tris‐SO4 pH 9.4, and 10 mm DTT (1.6 mL·g^−1^ of cell pellet) for 20 min at 30 °C. Cells were pelleted and washed with 1.2 m sorbitol and 20 mm KPi, pH 7.4 (1 mL·g^−1^ of cell pellet). In order to remove the cell wall, cells were resuspended in 1.2 m sorbitol and 20 mm KPi, pH 7.4 (5 mL·g^−1^ of cell pellet) in the presence of Zymolyase (3.5 mg·g^−1^ cell pellet) and incubated for 45 min at 30 °C, shaking. The spheroplasts were washed in the same buffer and homogenised on ice in 0.6 m sorbitol, 20 mm K‐MES pH 6.0, 1 mm PMSF with a glass homogeniser potter. Cell debris was removed by centrifugation at 1500 ***g*** and mitochondrial‐containing pellets were obtained by centrifuging supernatants at 12 000 ***g***. The pellets were homogenised using Teflon dounce, followed by a 1500 ***g*** centrifugation to obtain the supernatant. The crude mitochondrial pellet was collected by centrifugation at 12 000 ***g*** for 15 min, 4 °C. Next, mitochondria were loaded on Nycodenz gradients (Nycodenz 20%‐14.5%, 0.6 m sorbitol, 20 mm K‐MES pH 6.0) as previously described [25], and centrifuged for 60 min at 268 320 ***g***, 4 °C. Pure mitochondria were collected from the gradient interface and washed with 0.6 m sorbitol, 20 mm HEPES‐KOH pH 7.4. Finally, the protein concentration was adjusted to 20 mg·mL^−1^ in the previous buffer by also adding 10 mg·mL^−1^ of fatty‐acid free BSA. Mitochondria were snap frozen in liquid nitrogen and stored at −80 °C.

### Import into yeast mitochondria

^35^S‐Methionine radiolabelled precursor proteins were synthesised using the TNT SP6‐coupled transcription/translation kit (Promega, Madison, WI, USA) and plasmid‐vectors pSP64 containing the genes of interest. The radioactive material was imported in 50–100 μg of wild‐type, ΔTom70, Tim9ts or Tim22ts yeast mitochondria in the presence of 2 mm ATP and 2.5 mm NADH for 15 min at 30 °C. Mitochondria were then resuspended in 1.2 M sorbitol and 20 mm Hepes, pH 7.4, followed by a treatment with 0.05 mg·mL^−1^ trypsin to remove unimported material. In one sample 1% (vol/vol) Triton X‐100 was added as a control to completely solubilise the mitochondria. Trypsin inactivation was performed with 0.5 mg·mL^−1^ soybean trypsin inhibitor (SBTI) for 10 min on ice. Finally, mitochondria were spun down and resuspended in Laemmli sample buffer with β‐mercaptoethanol, analysed by SDS‐PAGE, and visualised by digital autoradiography (Molecular Dynamics) [53] as described in “Immunoblotting of HEK293 and yeast proteins.” The primary antibody mtHSP70 (Laboratory of Kostas Tokatlidis, [54], 1 : 20 000) was used as a housekeeping gene when immunoblotting yeast proteins and visualised with the Goat Anti‐Rabbit IgG (whole molecule)–Peroxidase secondary antibody (Sigma‐Aldrich Cat #: A6154, 1 : 10 000). To isolate specific stages of the carrier import pathway the following changes were made during import. To deplete ATP during import 25 U·mL^−1^ of apyrase was added to the reticulocyte lysate and incubated for 5 min prior to import. Meanwhile, mitochondria were treated with 20 mm of oligomycin for 5 min [29]. To deplete the membrane potential mitochondria were treated with 10 mm carbonyl cyanide m‐chlorophenyl hydrazone (CCCP). For the chase import experiment (Fig. 2B) precursor proteins were imported into mitochondria that were depleted of the membrane potential as above. Mitochondria were then spun (16 000 ***g***) and resuspended in fresh import buffer containing NADH to re‐establish the membrane potential [55]. The rest of the import process was carried out as above.

### Import into mammalian mitochondria

The radiolabelled precursor proteins were translated as above. Following this the precursor proteins were imported into 25 μg of isolated wild‐type HEK293 mitochondria for 30 s to 15 min at 37 °C [56]. The import buffer consisted of 250 mm sucrose, 5 mm magnesium acetate, 80 mm potassium acetate, 10 mm sodium succinate, 1 mm DTT, 5 mm ATP, 20 mm HEPES‐KOH, pH 7.4. To dissipate the membrane potential, 10 mm of CCCP was used. After import the mitochondria were spun (12 000 ***g***) and resuspended in fresh import buffer containing 0.05 mg·mL^−1^ trypsin. Trypsin was then inactivated with SBTI for 10 min on ice. Mitochondria were spun as above and resuspended in Laemmli sample buffer with β‐mercaptoethanol, analysed by SDS‐PAGE and visualised by digital autoradiography (Molecular Dynamics) as described in “Immunoblotting of HEK293 and yeast proteins.” Primary mouse antibody TFAM (Abnova Cat #: H00007019‐B01P, 1 : 1000) was used as the loading control and visualised with the Goat Anti‐Mouse IgG, H&L Chain Specific–Peroxidase secondary antibody (Sigma‐Aldrich Cat #: 401215, 1 : 10 000).

### Isolation of synaptosomes and TMT proteomics

Synaptosomes from brains of 1‐year old WT (three male, three female) and *Sfxn3*‐KO (three male, three female) mice were prepared as previously described [57]. Mice forebrains were homogenised in ice‐cold synaptosome isolation buffer (0.32 m Sucrose, 1 mm EDTA, 5 mm Tris‐HCl, pH 7.4). The homogenate was spun at 900 ***g*** for 10 min at 4 °C and the supernatant (S1) was removed to a new eppendorf. The pellet (P1) was resuspended in synaptosome isolation buffer and spun once again at 900 ***g*** for 10 min at 4 °C. The resulting supernatant (S1’) was added to the eppendorf containing S1. The pellet, instead, was labelled as the non‐synaptic fraction. The supernatant (S1 and S1’ combined) which contained the synaptosomes was spun at 20 000 ***g*** for 20 min at 4 °C. The resulting supernatant (S2) was discarded, and the pellet (P2) which contained the synaptosomes was kept.

Protein extraction was performed using Label‐free extraction buffer (100 mm Tris‐HCl, 4%(w/v) SDS, 1% Protease Inhibitor Cocktail (Roche, Cat #: 11836153001), pH 7.6). Extraction buffer was added to the sample to make an approximate 1 : 10 solution. The pellet containing synaptosomes was homogenised using a handheld homogeniser until producing a smooth homogenate. The homogenate was centrifuged at 20 000 ***g*** for 20 min at 4 °C, and the supernatant, which contained the soluble protein, was aspirated and placed in a new eppendorf. Following protein extraction from synaptosomes, a Micro BCA assay (Thermo Fisher Scientific) was performed to measure the concentration of protein obtained. After determining the concentration of protein in each sample, samples were pooled based on genotype, ensuring equal amounts of each sample were added to the WT or *Sfxn3*‐KO pools.

Proteomic processing (performed in collaboration with Fingerprints Proteomics Facility, University of Dundee) was begun by generating tryptic peptides of the protein samples via filter‐aided sample preparation (FASP). Peptides were then desalted and quantified prior to TMT labelling. After the TMT‐labelled peptides were dried down using a Speed Vac, they were fractionated using high pH fractionation, meaning they were separated based on their hydrophobic binding properties. The fractions were then pooled into a reduced number of groups ranging across the hydrophobicity spectrum. Quality control was performed on the fractions, and finally, underwent liquid chromatography‐tandem mass spectrometry (LC‐MS/MS) analysis. Data were analysed using the bioinformatics tool IPA and IPA was used to identify the top canonical pathways identified in proteins altered by ≥ 20% in *Sfxn3*‐KO mice. The complete proteomics dataset can be found at: https://doi.org/10.7488/ds/3068.

### Immunoblotting of mouse subcellular fractions

For mice brain mitochondria, protein extraction was performed using RIPA buffer + 1% proteinase inhibitor. The mitochondrial pellet was lysed using a 200 μL pipet and subsequently left on ice for 10 min. The sample was centrifuged at full speed for 10 min, and the supernatant containing the mitochondrial protein sample was collected into a new eppendorf. For mice brain synaptosomes, protein extraction was performed as described above, under “Isolation of synaptosomes and TMT proteomics.” Micro BCA Assays (Thermo Fisher Scientific) were performed to determine protein concentration. Once samples were prepared at the appropriate protein concentration with NuPage^®^ LDS Sample buffer 4X (Invitrogen, Waltham, MA, USA), samples were denatured at 70 °C for 10 min. SDS‐Page was then run on commercially produced precast NuPage 4–12% BisTris gradient gels (Thermo Fisher Scientific) and transferred onto PVDF membranes (Life Technologies, Carlsbad, CA, USA) using the iBlot 7‐Minute Blotting System (Life Technologies). REVERT Total Protein Stain (Licor, Lincoln, NE, USA) was used as a loading control for total protein after transfer. After rinsing off the REVERT Total Protein Stain, the membrane was placed in Odyssey blocking buffer (Licor) for 30 min and then incubated with the following primary antibodies overnight at 4 °C: CSPα (Enzo, Farmingdale, NY, USA, Cat #: ADI‐VAP‐SV003‐E, 1 : 2000), Uncleaved Caspase‐3 (Cell Signaling Technologies, Danvers, MA, USA, Cat #: 9662, 1 : 1000), Cytochrome C (Abcam, Cambridge, UK, Cat #: ab110325, 1 : 1000), OXPHOS cocktail (Abcam Cat #: ab110411, 1 : 1000), Rieske Fe‐S (A‐5) (Santa Cruz Biotechnology, Dallas, TX, USA, Cat #: sc‐271609, 1 : 1000), SHMT2 (Atlas Antibodies Cat #: HPA020549, 1 : 750), CHCHD3 (Proteintech Cat #: 25625‐1‐AP, 1 : 2000), Bcl‐2 (Proteintech Cat #: 12789‐1‐AP, 1 : 1000), Mitofusin 1 (Proteintech Cat #: 13798‐1‐AP, 1 : 2000) and NDUFS1 (Abcam Cat #: ab169540, 1 : 2000). After overnight incubation, the membrane was washed with 1x PBS and incubated with the following secondary antibodies for 1 h at room temperature: IRDye^®^ 800CW Donkey anti‐Rabbit IgG (H + L) (Licor Cat #: 926‐32213, 1 : 5000) and IRDye^®^ 800CW Donkey anti‐Mouse IgG (H + L) (Licor Cat #: 926‐32212, 1 : 5000). The membranes were then dried and imaged using the Odyssey Infrared Imaging System (Licor). Quantification and analysis of membranes were performed using image studio lite software (Licor).

### Brain histology and immunohistochemistry

Brains from 1‐year‐old *Sfxn3*‐KO and WT mice were dissected along the great longitudinal fissure, fixed in 4% paraformaldehyde (PFA) and cryopreserved using 30% sucrose. Brains were embedded in a 1 : 1 solution of optimal cutting temperature compound (OCT) and 30% sucrose and frozen within moulds on dry ice. Embedded brains were sectioned coronally on a cryostat at a thickness of 20 μm. Sections were placed onto Superfrost slides.

For histological analysis, Nissl bodies within neurons were stained with 0.2% cresyl fast violet (CFV) with 1% Acetic Acid. Sections were placed through increasing concentrations of ethanol, placed in xylene for 10 min and then rehydrated through decreasing concentrations of ethanol. Sections were then rinsed in water and stained with CFV for 1 min. After a final rinse in water, sections were left to dry overnight and coverslipped using DPX mountant. Sections were imaged using the PL Fluortar 2.5x/0.07 objective on the Leica DMRE epifluorescence microscope with Retiga 2000R (Qimaging, Surrey, Canada) Camera and acquired using the QCapture Pro v6.0 software (Qimaging). Individual images of brain sections were stitched together with Fiji to create single images of whole sections. Measurements of the width of the corpus callosum, the primary motor cortex, the primary somatosensory cortex, and the area of the dentate gyrus were performed blinded to the genotype using fiji software [58].

For immunohistochemical analysis, slides were thawed for 1 h at room temperate. The sections were washed in TBS, placed in a microwave safe contained with pre‐heated Citric Acid Based Antigen Unmasking Solution (Vector Laboratories, Burlingame, CA, USA, Cat #: H‐3300), and microwaved for 3 min at a cooking power of P10. After a quick wash with TBS, sections were permeabilised with 5% Triton X‐100 in PBS for 20 min and then washed 3 × 5 min with TBS. Sections were then blocked for 1 h in 5% normal donkey serum (Sigma‐Aldrich) in TBS at room temperature. Next, the sections were incubated with Iba1 goat polyclonal antibody (Abcam Cat #: ab5076, 1 : 500) prepared in TBS, overnight, at 4 °C, and washed 3 × 5‐min with TBS. They were then incubated for 90 min in Donkey anti‐Goat IgG (H + L) Secondary Antibody, 488 (AlexaFluor, Invitrogen, Cat #: A11055, 1 : 400) made in 1% normal donkey serum in TBS at room temperature and then stained with DAPI (Invitrogen) for 10 minutes. Finally, the sections were washed 3 × 5‐min with TBS and coverslipped with Mowiol. Once coverslipped, brain sections were imaged using the Plan Apochromat 20x/0.8 objective on the Zeiss LSM800 with Airy Scan confocal microscope and Z‐stack images were acquired using zen blue software (Zeiss, Cambourne, UK). Quantification of Iba1 was performed on sections where the dentate gyrus is first fully formed (Bregma −1.46). Quantification and analysis were performed blinded to the genotype using fiji software.

### *Drosophila* stock lines

Flies were raised on standard cornmeal food at 25 °C. The double transgenic lines were generated using the following stocks obtained from Bloomington *Drosophila* Stock Center: CG11739 (no. 15360 and 38230), P{UAS‐Hsap\SNCA.A30P} (no. 8147), P{UAS‐Hsap\SNCA.A53T} (no. 8148). P{5xUAS‐mCD8::GFP} control lines were obtained from Dr L Neukomm [59]. Double transgenic stock lines under UAS enhancer control were crossed with elav‐Gal4 driver strains. All fly lines used in this study were generated on the *Canton S* background. The *Drosophila* genotypes used in the experiment (Fig. 6D,E) were: w; 5xUAS‐mcD8::GFP/+; 5xUAS‐mcD8::GFP/+ (control); w; 5xUAS‐mcD8::GFP/+; UAS‐Snca^A30P^/+ (A30P PD model); w; 5xUAS‐mcD8::GFP/+; UAS‐Snca^A53T^/+ (A53T PD model); w; UAS‐Sfxn3^EY01545^/+; UAS‐Snca^A30P^/+ (A30P PD model with *Sfxn3* overexpression); w; UAS‐Sfxn3RNAi/+; UAS‐Snca^A30P^/+ (A30P PD model with *Sfxn3* RNAi); w; UAS‐Sfxn3^EY01545^/+; UAS‐Snca^A53T^/+ (A53T PD model with *Sfxn3* overexpression); w; UAS‐Sfxn3RNAi/+; UAS‐Snca^A53T^/+ (A30P PD model with *Sfxn3* RNAi).

### *Drosophila* immunohistochemistry

Adult brain dissections were performed as previously described [60]. Briefly, decapitated heads from 7‐week‐old flies were fixed in 4% formaldehyde in PTX (0.5% Triton X‐100, PBS) for 20 min, and washed five times for 2 min each in PTX. Dissected brains were fixed in 4% formaldehyde in PTX for 10 min, washed five times for 2 min each in PTX and then incubated with Tyrosine Hydroxylase (Merck, Kenilworth, NJ, USA, Cat #: Ab152, 1 : 400) and Cy™3 AffiniPure Goat Anti‐Rabbit IgG (H + L) (Jackson Immunoresearch, West Grove, PA, USA, Cat #: 111‐165‐003, 1 : 200) to detect dopaminergic neurons: Brains were then washed three times for 10 min each at room temperature and mounted in Vectashield for microscopy. Once coverslipped, *Drosophila* brains were imaged using the HC PL APO CS2 63x/1.4 objective using a zoom factor of 3.5 on the Leica TCS SP8 inverted confocal microscope. Z‐stack images were acquired using LAS X Navigator software.

### Quantification and statistical analysis

Immunoblotting experiment quantification was performed with imagestudiolite v5.2.5 and microscope image quantification was performed with fiji v2.1.0 [58]. All statistical analyses were performed using graphpad prism v8.2.0 (GraphPad Software Inc, San Diego, CA, USA). The statistical tests performed, their significance levels and the value of *n* are referred to in the corresponding figure legends. Statistical significance was considered to be *P* < 0.05 and all graphs are plotted as mean ± SEM.

## Conflict of interest

The authors declare no conflict of interest.

## Author contributions

LML, MET, KT, LCG, TMW and THG designed and coordinated the study. LML and MET, RE, RAK, LCG, SLE, DvdH, HC, YTH, EJNG, AALM and DJL contributed to data collection and analysis. KT, TMW and THG contributed to data analysis. LML, MET and THG wrote the manuscript to which RE, KT and TMW contributed.

### Peer review

The peer review history for this article is available at https://publons.com/publon/10.1111/febs.16377.

## Data Availability

The complete proteomics datasets generated in this study are freely available to download from https://doi.org/10.7488/ds/3068. All other raw data will be made available, upon reasonable request, by the corresponding author.

## References

[1] Bae JR , Kim SH . Synapses in neurodegenerative diseases. BMB Rep. 2017;50:237–46. 28270301 10.5483/BMBRep.2017.50.5.038 PMC5458673

[2] Sun J , Harrington MA . The alteration of intrinsic excitability and synaptic transmission in lumbar spinal motor neurons and interneurons of severe spinal muscular atrophy mice. Front Cell Neurosci. 2019;13:15. 30792629 10.3389/fncel.2019.00015 PMC6374350

[3] Koch S , Molchanova SM , Wright AK , Edwards A , Cooper JD , Taira T , et al. Morphologic and functional correlates of synaptic pathology in the cathepsin D knockout mouse model of congenital neuronal ceroid lipofuscinosis. J Neuropathol Exp Neurol. 2011;70:1089–96. 22082660 10.1097/NEN.0b013e318238fc28 PMC3242052

[4] Kashyap G , Bapat D , Das D , Gowaikar R , Amritkar RE , Rangarajan G , et al. Synapse loss and progress of Alzheimer’s disease ‐A network model. Sci Rep. 2019;9:6555. 31024073 10.1038/s41598-019-43076-y PMC6484103

[5] Nguyen M , Krainc D . LRRK2 phosphorylation of auxilin mediates synaptic defects in dopaminergic neurons from patients with Parkinson's disease. Proc Natl Acad Sci USA. 2018;115:5576–81. 29735704 10.1073/pnas.1717590115 PMC6003526

[6] Phan J‐A , Stokholm K , Zareba‐Paslawska J , Jakobsen S , Vang K , Gjedde A , et al. Early synaptic dysfunction induced by α‐synuclein in a rat model of Parkinson's disease. Sci Rep. 2017;7:6363. 28743955 10.1038/s41598-017-06724-9 PMC5526979

[7] Grünewald B , Lange MD , Werner C , O’Leary A , Weishaupt A , Popp S , et al. Defective synaptic transmission causes disease signs in a mouse model of juvenile neuronal ceroid lipofuscinosis. eLife. 2017;6:e28685. 29135436 10.7554/eLife.28685 PMC5724993

[8] Bridi JC , Hirth F . Mechanisms of α‐synuclein induced synaptopathy in Parkinson's disease. Front Neurosci. 2018;12:80. 29515354 10.3389/fnins.2018.00080 PMC5825910

[9] Zhang P , Park HJ , Zhang J , Junn E , Andrews RJ , Velagapudi SP , et al. Translation of the intrinsically disordered protein α‐synuclein is inhibited by a small molecule targeting its structured mRNA. Proc Natl Acad Sci USA. 2020;117:1457–67. 31900363 10.1073/pnas.1905057117 PMC6983430

[10] Amorim IS , Graham LC , Carter RN , Morton NM , Hammachi F , Kunath T , et al. Sideroflexin 3 is an α‐synuclein‐dependent mitochondrial protein that regulates synaptic morphology. J Cell Sci. 2017;130:325–31. 28049716 10.1242/jcs.194241 PMC5278670

[11] Li X , Han D , Kam RKT , Guo X , Chen M , Yang Y , et al. Developmental expression of sideroflexin family genes in Xenopus embryos. Dev Dyn. 2010;239:2742–7. 20737508 10.1002/dvdy.22401

[12] Fleming MD , Campagna DR , Haslett JN , Trenor Iii CC , Andrews NC . A mutation in a mitochondrial transmembrane protein is responsible for the pleiotropic hematological and skeletal phenotype of flexed‐tail (f/f) mice. Genes Dev. 2001;15:652–7. 11274051 10.1101/gad.873001 PMC312659

[13] Kory N , Wyant GA , Prakash G , uit de Bos J , Bottanelli F , Pacold ME , et al. SFXN1 is a mitochondrial serine transporter required for one‐carbon metabolism. Science. 2018;362:eaat9528. 30442778 10.1126/science.aat9528 PMC6300058

[14] Fuller HR , Hurtado ML , Wishart TM , Gates MA . The rat striatum responds to nigro‐striatal degeneration via the increased expression of proteins associated with growth and regeneration of neuronal circuitry. Proteome Sci. 2014;12:1–16. 24834013 10.1186/1477-5956-12-20 PMC4021461

[15] Simunovic F , Yi M , Wang Y , Macey L , Brown LT , Krichevsky AM , et al. Gene expression profiling of substantia nigra dopamine neurons: further insights into Parkinson's disease pathology. Brain. 2009;132:1795–809. 19052140 10.1093/brain/awn323 PMC2724914

[16] Charbonnier‐Beaupel F , Malerbi M , Alcacer C , Tahiri K , Carpentier W , Wang C , et al. Gene expression analyses identify Narp contribution in the development of L‐DOPA‐induced dyskinesia. J Neurosci. 2015;35:96–111. 25568106 10.1523/JNEUROSCI.5231-13.2015 PMC6605247

[17] Chen B , Aredo B , Ding Y , Zhong X , Zhu Y , Zhao CX , et al. Forward genetic analysis using OCT screening identifies Sfxn3 mutations leading to progressive outer retinal degeneration in mice. Proc Natl Acad Sci USA. 2020;117:12931–42. 32457148 10.1073/pnas.1921224117 PMC7293615

[18] Fujiki Y , Hubbard L , Fowler S , Lazarow PB . Isolation of intracellular membranes by means of sodium carbonate treatment: application to endoplasmic reticulum. J Cell Biol. 1982;93:97–102. 7068762 10.1083/jcb.93.1.97 PMC2112113

[19] Ngo HB , Lovely GA , Phillips R , Chan DC . Distinct structural features of TFAM drive mitochondrial DNA packaging versus transcriptional activation. Nat Commun. 2014;5:3077. 24435062 10.1038/ncomms4077 PMC3936014

[20] Fisher RP , Lisowsky T , Parisi MA , Clayton DA . DNA wrapping and bending by a mitochondrial high mobility group‐like transcriptional activator protein. J Biol Chem. 1992;267:3358–67. 1737790

[21] Rojo M , Legros F , Chateau D , Lombès A . Membrane topology and mitochondrial targeting of mitofusins, ubiquitous mammalian homologs of the transmembrane GTPase Fzo. J Cell Sci. 2002;115:1663–74. 11950885 10.1242/jcs.115.8.1663

[22] Darshi M , Mendiola VL , Mackey MR , Murphy AN , Koller A , Perkins GA , et al. ChChd3, an inner mitochondrial membrane protein, is essential for maintaining Crista integrity and mitochondrial function. J Biol Chem. 2011;286:2918–32. 21081504 10.1074/jbc.M110.171975 PMC3024787

[23] John GB , Shang Y , Li L , Renken C , Mannella CA , Selker JML , et al. The mitochondrial inner membrane protein mitofilin controls cristae morphology. Mol Biol Cell. 2005;16:1543–54. 15647377 10.1091/mbc.E04-08-0697 PMC551514

[24] Yoshikumi Y , Mashima H , Ueda N , Ohno H , Suzuki J , Tanaka S , et al. Roles of CTPL/Sfxn3 and Sfxn family members in pancreatic islet. J Cell Biochem. 2005;95:1157–68. 15864810 10.1002/jcb.20481

[25] Kritsiligkou P , Chatzi A , Charalampous G , Mironov A , Grant CM , Tokatlidis K . Unconventional targeting of a thiol peroxidase to the mitochondrial intermembrane space facilitates oxidative protein folding. Cell Rep. 2017;18:2729–41. 28297675 10.1016/j.celrep.2017.02.053 PMC5368413

[26] Truscott KN , Pfanner N . Import of proteins into mitochondria. Biol Chem. 1999;380:1151–6. 10595577 10.1515/BC.1999.146

[27] Wiedemann N , Pfanner N . Mitochondrial machineries for protein import and assembly. Annu Rev Biochem. 2017;86:685–714. 28301740 10.1146/annurev-biochem-060815-014352

[28] Pfanner N , Tropschug M , Neupert W . Mitochondrial protein import: nucleoside triphosphates are involved in conferring import‐competence to precursors. Cell. 1987;49:815–23. 2884042 10.1016/0092-8674(87)90619-2

[29] Ryan MT , Müller H , Pfanner N . Functional staging of ADP/ATP carrier translocation across the outer mitochondrial membrane. J Biol Chem. 1999;274:20619–27. 10400693 10.1074/jbc.274.29.20619

[30] Koehler CM , Merchant S , Oppliger W , Schmid K , Jarosch E , Dolfini L , et al. Tim9p, an essential partner subunit of Tim10p for the import of mitochondrial carrier proteins. EMBO J. 1998;17:6477–86. 9822593 10.1093/emboj/17.22.6477 PMC1170995

[31] Graham LC , Eaton SL , Brunton PJ , Atrih A , Smith C , Lamont DJ , et al. Proteomic profiling of neuronal mitochondria reveals modulators of synaptic architecture. Mol Neurodegener. 2017;12:77. 29078798 10.1186/s13024-017-0221-9 PMC5659037

[32] Mazzara PG , Muggeo S , Luoni M , Massimino L , Zaghi M , Valverde PTT , et al. Frataxin gene editing rescues Friedreich’s ataxia pathology in dorsal root ganglia organoid‐derived sensory neurons. Nat Commun. 2020;11:1–18. 32826895 10.1038/s41467-020-17954-3 PMC7442818

[33] Stehling O , Netz DJA , Niggemeyer B , Rösser R , Eisenstein RS , Puccio H , et al. Human Nbp35 is essential for both cytosolic iron‐sulfur protein assembly and iron homeostasis. Mol Cell Biol. 2008;28:5517–28. 18573874 10.1128/MCB.00545-08 PMC2519719

[34] Zhang Z , Hou L , Song JL , Song N , Sun YJ , Lin X , et al. Pro‐inflammatory cytokine‐mediated ferroportin down‐regulation contributes to the nigral iron accumulation in lipopolysaccharide‐induced Parkinsonian models. Neuroscience. 2014;257:20–30. 24183966 10.1016/j.neuroscience.2013.09.037

[35] You LH , Li F , Wang L , Zhao SE , Wang SM , Zhang LL , et al. Brain iron accumulation exacerbates the pathogenesis of MPTP‐induced Parkinson's disease. Neuroscience. 2015;284:234–46. 25301748 10.1016/j.neuroscience.2014.09.071

[36] Matak P , Matak A , Moustafa S , Aryal DK , Benner EJ , Wetsel W , et al. Disrupted iron homeostasis causes dopaminergic neurodegeneration in mice. Proc Natl Acad Sci USA. 2016;113:3428–35. 26929359 10.1073/pnas.1519473113 PMC4822577

[37] Biagioli M , Pinto M , Cesselli D , Zaninello M , Lazarevic D , Roncaglia P , et al. Unexpected expression of α‐ and β‐globin in mesencephalic dopaminergic neurons and glial cells. Proc Natl Acad Sci USA. 2009;106:15454–9. 19717439 10.1073/pnas.0813216106 PMC2732704

[38] Braymer JJ , Lill R . Iron–sulfur cluster biogenesis and trafficking in mitochondria. J Biol Chem. 2017;292:12754–63. 28615445 10.1074/jbc.R117.787101 PMC5546016

[39] Wachnowsky C , Fidai I , Cowan JA . Iron‐sulfur cluster biosynthesis and trafficking‐impact on human disease conditions. Metallomics. 2018;10:9–29. 29019354 10.1039/c7mt00180k PMC5783746

[40] Isaya G . Mitochondrial iron‐sulfur cluster dysfunction in neurodegenerative disease. Front Pharmacol. 2014;5:1–7. 24624085 10.3389/fphar.2014.00029 PMC3939683

[41] Chandra S , Gallardo G , Fernández‐Chacón R , Schlüter OM , Südhof TC . α‐Synuclein cooperates with CSPα in preventing neurodegeneration. Cell. 2005;123:383–96. 16269331 10.1016/j.cell.2005.09.028

[42] Fontaine SN , Zheng D , Sabbagh JJ , Martin MD , Chaput D , Darling A , et al. DnaJ/Hsc70 chaperone complexes control the extracellular release of neurodegenerative‐associated proteins. EMBO J. 2016;35:1537–49. 27261198 10.15252/embj.201593489 PMC4946142

[43] McIlwain DR , Berger T , Mak TW . Caspase functions in cell death and disease. Cold Spring Harb Perspect Biol. 2013;5:a008656. 23545416 10.1101/cshperspect.a008656 PMC3683896

[44] Mohite GM , Dwivedi S , Das S , Kumar R , Paluri S , Mehra S , et al. Parkinson's disease associated α‐synuclein familial mutants promote dopaminergic neuronal death in *Drosophila melanogaster* . ACS Chem Neurosci. 2018;9:2628–38. 29906099 10.1021/acschemneuro.8b00107

[45] Botella JA , Bayersdorfer F , Schneuwly S . Superoxide dismutase overexpression protects dopaminergic neurons in a Drosophila model of Parkinson's disease. Neurobiol Dis. 2008;30:65–73. 18243716 10.1016/j.nbd.2007.11.013

[46] Bachiller S , Jiménez‐Ferrer I , Paulus A , Yang Y , Swanberg M , Deierborg T , et al. Microglia in neurological diseases: a road map to brain‐disease dependent‐inflammatory response. Front Cell Neurosci. 2018;12:488. 30618635 10.3389/fncel.2018.00488 PMC6305407

[47] Wishart TM , Paterson JM , Short DM , Meredith S , Robertson KA , Sutherland C , et al. Differential proteomics analysis of synaptic proteins identifies potential cellular targets and protein mediators of synaptic neuroprotection conferred by the slow Wallerian degeneration (Wlds) gene. Mol Cell Proteomics. 2007;6:1318–30. 17470424 10.1074/mcp.M600457-MCP200 PMC2225590

[48] Minton DR , Nam M , McLaughlin DJ , Shin J , Bayraktar EC , Alvarez SW , et al. Serine catabolism by SHMT2 is required for proper mitochondrial translation initiation and maintenance of formylmethionyl tRNAs denise. Mol Cell. 2018;69:610–21. 29452640 10.1016/j.molcel.2018.01.024 PMC5819360

[49] Mon EE , Wei F‐Y , Ahmad RNR , Yamamoto T , Moroishi T , Tomizawa K . Regulation of mitochondrial iron homeostasis by sideroflexin 2. J Physiol Sci. 2019;69:359–73. 30570704 10.1007/s12576-018-0652-2 PMC6373408

[50] Paul BT , Tesfay L , Winkler CR , Torti FM , Torti SV . Sideroflexin 4 affects Fe‐S cluster biogenesis, iron metabolism, mitochondrial respiration and heme biosynthetic enzymes. Sci Rep. 2019;9:19634. 31873120 10.1038/s41598-019-55907-z PMC6928202

[51] Terzenidou ME , Segklia A , Kano T , Papastefanaki F , Karakostas A , Charalambous M , et al. Novel insights into SLC25A46‐related pathologies in a genetic mouse model. PLoS Genet. 2017;13:e1006656. 28376086 10.1371/journal.pgen.1006656 PMC5380310

[52] Wagner K , Gebert N , Guiard B , Brandner K , Truscott KN , Wiedemann N , et al. The assembly pathway of the mitochondrial carrier translocase involves four preprotein translocases. Mol Cell Biol. 2008;28:4251–60. 18458057 10.1128/MCB.02216-07 PMC2447139

[53] Sideris DP , Petrakis N , Katrakili N , Mikropoulou D , Gallo A , Ciofi‐Baffoni S , et al. A novel intermembrane space‐targeting signal docks cysteines onto Mia40 during mitochondrial oxidative folding. J Cell Biol. 2009;187:1007–22. 20026652 10.1083/jcb.200905134 PMC2806287

[54] Purohit PK , Edwards R , Tokatlidis K , Saini N . MiR‐195 regulates mitochondrial function by targeting mitofusin‐2 in breast cancer cells. RNA Biol. 2019;16:918–29. 30932749 10.1080/15476286.2019.1600999 PMC6546347

[55] Ellenrieder L , Dieterle MP , Doan KN , Mårtensson CU , Floerchinger A , Campo ML , et al. Dual role of mitochondrial porin in metabolite transport across the outer membrane and protein transfer to the inner membrane. Mol Cell. 2019;73:1056–65. 30738704 10.1016/j.molcel.2018.12.014

[56] Kang Y , Stroud DA , Baker MJ , De Souza DP , Frazier AE , Liem M , et al. Sengers syndrome‐associated mitochondrial acylglycerol kinase is a subunit of the human TIM22 protein import complex. Mol Cell. 2017;67:457–70. 28712726 10.1016/j.molcel.2017.06.014

[57] Wishart TM , Rooney TM , Lamont DJ , Wright AK , Morton AJ , Jackson M , et al. Combining comparative proteomics and molecular genetics uncovers regulators of synaptic and axonal stability and degeneration in vivo. PLoS Genet. 2012;8:e1002936. 22952455 10.1371/journal.pgen.1002936 PMC3431337

[58] Schindelin J , Arganda‐Carreras I , Frise E , Kaynig V , Longair M , Pietzsch T , et al. Fiji: an open‐source platform for biological‐image analysis. Nat Methods. 2012;9:676–82. 22743772 10.1038/nmeth.2019 PMC3855844

[59] Neukomm LJ , Burdett TC , Seeds AM , Hampel S , Coutinho‐Budd JC , Farley JE , et al. Axon death pathways converge on axundead to promote functional and structural axon disassembly. Neuron. 2017;95:78–91. 28683272 10.1016/j.neuron.2017.06.031

[60] Vosshall LB , Wong AM , Axel R . An olfactory sensory map in the fly brain. Cell. 2000;102:147–59. 10943836 10.1016/s0092-8674(00)00021-0

[61] Truscott KN , Wiedemann N , Rehling P , Müller H , Meisinger C , Pfanner N , et al. Mitochondrial import of the ADP/ATP carrier: the essential TIM complex of the intermembrane space is required for precursor release from the TOM complex. Mol Cell Biol. 2002;22:7780–9. 12391147 10.1128/MCB.22.22.7780-7789.2002 PMC134741

[62] Rampelt H , Sucec I , Bersch B , Horten P , Perschil I , Martinou JC , et al. The mitochondrial carrier pathway transports non‐canonical substrates with an odd number of transmembrane segments. BMC Biol. 2020;18:2. 31907035 10.1186/s12915-019-0733-6 PMC6945462

[63] Mao Z , Davis RL . Eight different types of dopaminergic neurons innervate the Drosophila mushroom body neuropil: anatomical and physiological heterogeneity. Front Neural Circuits. 2009;3:1–17. 19597562 10.3389/neuro.04.005.2009 PMC2708966

